# Emergent properties of a computational model of tumour growth

**DOI:** 10.7717/peerj.2176

**Published:** 2016-06-29

**Authors:** Pan Pantziarka

**Affiliations:** The George Pantziarka TP53 Trust, London, United Kingdom; Anticancer Fund, Brussels, Belgium

**Keywords:** Cancer, Evolution, Computational model, Carcinogenesis, Tissue organisation field theory, Somatic mutation theory, Modelling, Genetic algorithm, Agent-based model, Tumour growth

## Abstract

While there have been enormous advances in our understanding of the genetic drivers and molecular pathways involved in cancer in recent decades, there also remain key areas of dispute with respect to fundamental theories of cancer. The accumulation of vast new datasets from genomics and other fields, in addition to detailed descriptions of molecular pathways, cloud the issues and lead to ever greater complexity. One strategy in dealing with such complexity is to develop models to replicate salient features of the system and therefore to generate hypotheses which reflect on the real system. A simple tumour growth model is outlined which displays emergent behaviours that correspond to a number of clinically relevant phenomena including tumour growth, intra-tumour heterogeneity, growth arrest and accelerated repopulation following cytotoxic insult. Analysis of model data suggests that the processes of cell competition and apoptosis are key drivers of these emergent behaviours. Questions are raised as to the role of cell competition and cell death in physical cancer growth and the relevance that these have to cancer research in general is discussed.

## Introduction

Tumour growth is a complex process characterised by multi-scale phenomena involving both cancer and non-cancer cell populations. Where previously our focus was directed primarily at the activities of the cancer cell populations, once conceptualised as a single homogeneous mass, our increased understanding of cancer biology now incorporates a more nuanced evolutionary or ecological view of cancer growth (Gatenby, Gillies & Brown, 2011; Kareva, 2011). Key elements of this view of cancer as an evolutionary system are a focus on the genetic heterogeneity of tumour cell populations (Fisher, Pusztai & Swanton, 2013; De Sousa E Melo et al., 2013), the importance of the tumour microenvironment and the cross-talk between cancer and non-cancer cell populations (Allen & Louise Jones, 2011; Hanahan & Coussens, 2012; Quail & Joyce, 2013). A concern among some investigators is that, in the absence of an evolutionary understanding of population dynamics in cancer, therapeutic interventions may be doomed to failure (Silva & Gatenby, 2010; Tian et al., 2011; Gillies, Verduzco & Gatenby, 2012). In other cases there is interest in understanding the role of the microenvironment in the process of cancer initiation (Pantziarka, 2015) or the metastatic cascade (Psaila et al., 2007; Barcellos-Hoff, Lyden & Wang, 2013).

More fundamentally, there are also competing theoretical views of cancer at the most basic level. The predominant view of cancer—termed the somatic mutation theory (SMT)—is that it is a disease caused, and then driven, by genetic mutations in cells. An alternative view—termed the tissue-organisation field theory (TOFT)—views cancer as a disease caused by tissue dysfunction, development gone astray, with genetic changes not as the drivers but as a consequence of the disease. A number of recent publications outline these competing views of cancer (Baker, 2014; Bizzarri & Cucina, 2014; Sonnenschein et al., 2014).

A challenge to all fundamental theories of cancer is to incorporate the vast array of new data that molecular biology has afforded to the researcher. The literature expands exponentially as we develop the tools to probe ever deeper into cellular structures, signalling pathways and the large data volumes generated by the various ‘omics.’ Against this backdrop of ever greater detail it is becoming harder to integrate the data into a coherent ‘big picture.’ Robert Weinberg makes the point that we are going full circle—from an initially complex picture of disjointed phenomenological facts to simplifying models arising from the revolution in molecular biology and back to a picture of endless complexity again (Weinberg, 2014). The impacts of this lack of progress are ultimately felt in the clinic, where, with a few significant exceptions, progress in developing treatments has significantly slowed in recent years (Jalali, Mittra & Badwe, 2016).

Computational models can provide ideal platforms for developing conceptual understanding of complex biological systems (Saetzler, Sonnenschein & Soto, 2011; Janes & Lauffenburger, 2013). A range of techniques are available to build such software models of cancer growth specifically to explore evolutionary or ecological hypotheses at an abstract and non-physiological level, including techniques from evolutionary game theory (Basanta et al., 2008; Krzeslak & Swierniak, 2014) and machine learning (Gerlee, Basanta & Anderson, 2011).

For example Ribba et al. (2004) created a hybrid cellular automaton model which aimed to replicate some features of CHOP therapy for Non-Hodgkin’s Lymphoma (NHL). The model was calibrated in such a way as to make specific predictions as to the response of NHL cells to treatment with the chemotherapeutic drug doxorubicin. Gerlee & Anderson (2007) developed an evolutionary hybrid cellular automaton model of solid tumour growth to investigate the impact of tissue oxygen concentration on the growth and evolutionary dynamics of a tumour. A key aspect of this model was the calibration of parameters with physically relevant data in terms of oxygen and glucose consumption rates, time estimates for cellular proliferation and so on. Enderling and colleagues have developed a series of hybrid cellular automaton models which include both qualitative and quantitative results related to cancer stem cell theory and tumour growth (Enderling, Hlatky & Hahnfeldt, 2009; Enderling & Hahnfeldt, 2011; Poleszczuk & Enderling, 2016). Closer in intent to this work was the genetic algorithm model developed by Gerlee, Basanta & Anderson (2011) to investigate the evolution of homeostatic tissue in a two-dimensional monolayer system.

NEATG (Non-physiological Evolutionary Algorithm for Tumour Growth) is a simple software model of tumour growth which models cell-to-cell and tissue-level interactions and population dynamics under different evolutionary scenarios. Furthermore the platform is structured such that anti-tumour interventions can also be modelled within these different scenarios. A number of scenarios are explored in this paper, including the simulation of cellular response to homeostasis, stress conditions, nutrient deprivation and cytotoxic intervention. The approach in this work is primarily qualitative rather than quantitative and does not depend on calibration to physical tumour growth models.

While computational models enable the construction of *in silico* experiments involving biological systems, they differ from traditional mathematical models (differential and other equation-based systems) in that the model itself is encoded in computer code, input/output file formats, configuration files etc. Therefore, it is important in reporting on such a model that there is exposition not just of the algorithmic details but also an exploration of how the model behaves at different stages, of results with differing inputs, the modelling of different scenarios and so on. Therefore the ‘Results’ of this work presents a significant level of detail in the hope that we can lessen the degree of opacity.

## Methods

NEATG is implemented as a hybrid model incorporating elements from both genetic algorithms and cellular automata. It is dual scale, non-deterministic and represents both cell-level and tissue-level behaviour. It is coded in the Java programming language.

### Grid or tissue-level

The tissue-level is represented as a rectangular grid, with each grid element containing a set of modelled cells, which may be Malignant or Normal. The relative proportion of Normal and Malignant cells in a grid element determines the state of that grid element. These states are:

\begin{eqnarray*}E=\{\text{Normal, Majority Normal, Majority Malignant, Tumour, Necrotic}\}. \end{eqnarray*}

**Figure 1:**
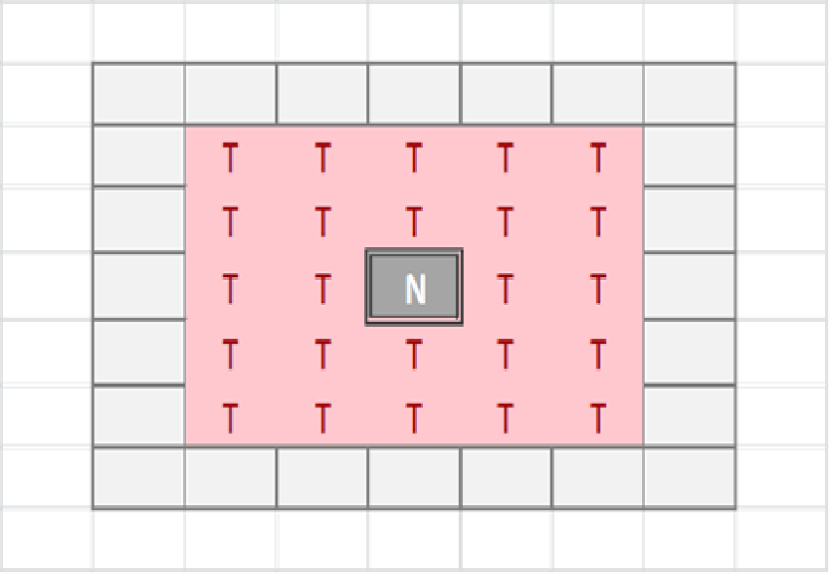
Moore neighbourhood of radius 2.

Transition of a grid element from one state to another takes place at every clock tick (generation) and is determined by the proportions of different cell populations within that element, but also by the state of neighbouring grid elements. Grid elements which are in the Tumour state (that is, they do not have any Normal cells within them) can transition to a Necrotic state if they are surrounded by an extended neighbourhood which consists exclusively of other Tumour grid elements. By default this is a Moore neighbourhood of radius 2 (see Fig. 1), though this is a configurable model parameter. This Necrotic state is designed to model cellular compartments within solid tumours in which a high rate of hypoxia and a low level of nutrient availability lead to high levels of cellular necrosis.

Grid elements in the Necrotic state are suspended and do not take part in further computational activity unless the neighbouring grid population changes, in which case the Necrotic state reverts to Tumour.

Each grid element is populated with an initial, optimum population of Normal cells. The size of this optimum population is a model input parameter. The size of the population can vary over time and can increase to a defined maximum value, termed the carrying capacity, after which cellular competition takes place (as described below).

Each grid element receives as input a Nutrient, represented as an integer value, and a set of Gene Factors, represented as real values. The number of Gene Factors is equal to the number of genes in the cell structure. The Nutrient score can be loosely interpreted as a combination of oxygen and cellular nutrients (e.g., glucose), while the Gene Factors may be viewed as generic growth factors required for cellular growth and survival.

The grid element has a distribution function to compute the share of Nutrient (*DN*) assigned to each cell in its population of *P* cells based on the relative demand represented by the Nutrient Target values *T* for each cell:

(1) \begin{eqnarray*}{\mathit{DN}}_{i}= \frac{{T}_{i}}{\sum _{p=1}^{P}{T}_{p}} .\end{eqnarray*}

Similarly the Gene Factor values which are inputs into each grid element are distributed to each cell according to the transfer function based on the Gene Targets (*G*):

(2) \begin{eqnarray*}{\mathit{DG}}_{i}= \frac{{G}_{i}}{\sum _{p=1}^{P}{G}_{p}} .\end{eqnarray*}

The distribution functions in Eqs. (1) and (2) are designed to share resources within a given grid element based on relative demand in order to reflect the levels of avidity in individual cells. In real cells this avidity is controlled via the glucose transporter proteins Glut1–Glut3, for example.

### Cell level

There are two types of cell in this model, Normal and Malignant, with the same internal structure regardless of type. While the structure is the same the behaviour is type-dependent during cell division, as will be shown later. Crucially, the Malignant cell has non-zero values for Mutation and Invasion rates, whereas for the Normal cells these values are set to zero.

Each cell is a data structure that encodes a Genome and an internal clock. The internal clock, implemented as an integer value, counts down from a maximum value, known as the Lifetime, to zero. Cell division is initiated when the clock reaches zero—Lifetime is therefore analogous to the length of time for the cell cycle. When the system is first instantiated each cell is initialised with an internal clock value that is equal to a random integer between the Lifetime and zero. In the experiments that follow, a Lifetime of 100 generations has been used as this simplifies the numerical analysis and empirical testing showed that it produced robust results in different scenarios. Shorter Lifetimes accelerated rates of cell proliferation, and longer values slowed growth rates however the qualitative results were similar in all cases. At initiation all cells have the same Lifetime, though this is heritable and mutable and therefore subject to evolutionary adaptation over time.

The Genome is a set of *N* genes, which are defined by a Target and a Tolerance, both represented as real numbers. The Genome is defined as:

\begin{eqnarray*}G=\{(\text{Target}0,\text{Gene Tolerance}0)\ldots (\mathrm{Target}N,\text{Tolerance}N)\}. \end{eqnarray*}

While the size of the genome is configurable, the default value of *N* is 3 in the experiments presented in this work. Empirical testing indicated that three genes were sufficient to illustrate genetic evolution and diversity. Increasing the value of *N* increased the run-time of the system but did not otherwise produce much change in the major output characteristics such as growth in Malignant cell numbers, measures of genetic heterogeneity etc. Decreasing *N* improved performance somewhat but with reduced scope for genetic evolution to take place.

The Gene Target is the optimum level of the corresponding Gene Factor that exists in the grid environment, and the Tolerance defines a band of tolerable values on either side of the Target that is the healthy range for that gene. Real cells are able to survive variations in nutrients, growth factors and so on; the Target and Tolerance mechanism is therefore a mechanism to allow modelled cells to similarly survive in varying conditions. Gene health is therefore defined as a Boolean value which evaluates as True when the Gene Factor is within the desired range, or False if the Gene Factor is above or below the tolerable range:

(3) \begin{eqnarray*}\text{Health}=(\text{Gene Factor}\lt (\text{Gene Target}+\text{Gene Tolerance}))\hspace*{1em}\mathrm{{\XMLAMP} }\hspace*{1em}(\text{Gene Factor}\gt (\text{Gene Target}-\text{Gene Tolerance})).\end{eqnarray*}

In addition to flagging health status, Genes are also used as a mechanism for the cell to influence the local grid environment. This is a simple feedback mechanism by which each cell attempts to alter the local environment in order to achieve the level of Gene Factor required for its own optimum health. The expression function is:

(4) \begin{eqnarray*}E=1-{e}^{-(T-F)}\end{eqnarray*}

where *T* is the Gene Target value and *F* exogenously supplied Factor.

The actual level of Gene Factor available in each Grid Element is calculated as the sum of the exogenously supplied Factor, which is an input parameter in the model, and the sum of the expression values from each cell in that grid element.

Additional components of the cell are the Nutrient Target and a Nutrient Rate, which represent the demand for nutrient and the rate at which nutrient is consumed respectively. Nutrient which is not consumed is stored in the Nutrient Store. Each cell also has a Mutation Rate and an Invasion Rate, which are used when cell division is necessitated for Malignant cells.

Cells can exist in a number of states:

\begin{eqnarray*}S=\{\text{HEALTHY, DIVIDING, APOPTOTIC, TO_BE_CLEARED, NECROTIC}\}. \end{eqnarray*}

Note that the cell state of Healthy implies viability, rather than whether a cell is Normal or Malignant.

At every clock tick the health status of each cell is assessed and the cell clock decremented according to the state of health. A healthy cell, with adequate Nutrient and Gene Factors, will decrease the cell clock by 1. Each unhealthy gene will also decrement the cell clock by one. A cell that has a value of zero for Nutrient store will have the cell clock set to zero because it is unable to meet its metabolic requirements and must therefore transition from a Healthy state.

All cells undergo a similar cell cycle. A cell starts as Healthy and undergoes a number of iterations (clock ticks) in which nutrient and gene factors are processed, the cell clock decreases at rates that depend on how well the cell is adapted to the local grid environment defined by the available Nutrient and Gene Factors. When the cell clock or nutrient store reaches zero the cell changes state according to the following cycle:

\begin{eqnarray*}\text{Healthy}\gt \text{Dividing}\gt \text{Apoptotic}\gt \text{To Be Cleared}. \end{eqnarray*}

**Figure 2:**
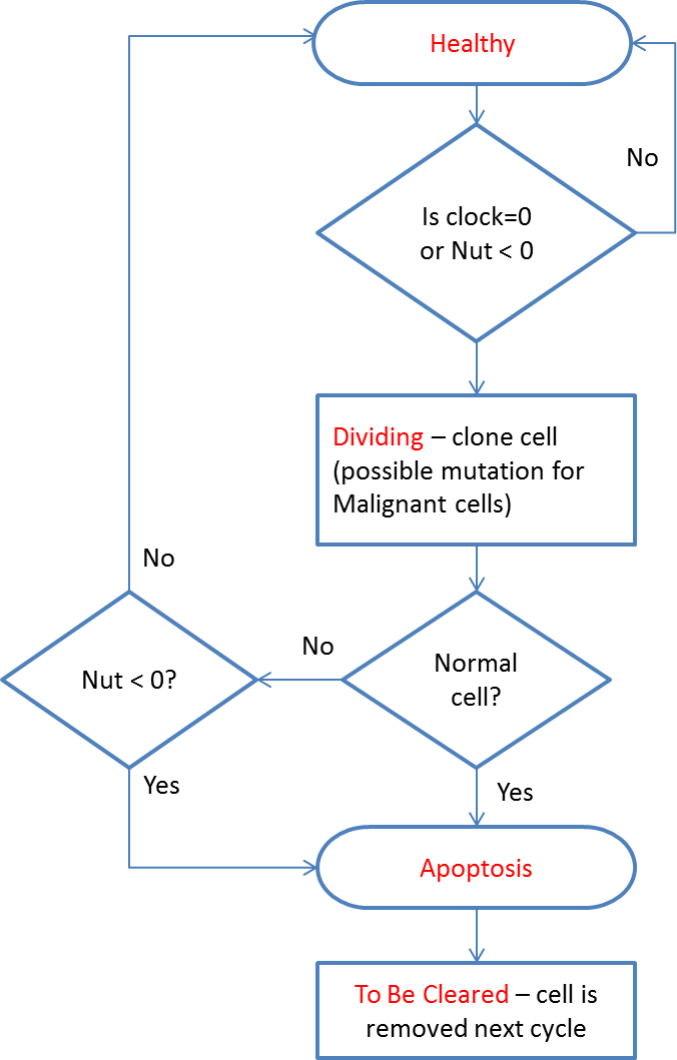
Cell cycle algorithm.

The cell cycle algorithm is shown in Fig. 2 (note that cell states are indicated in red). Cells that are flagged as To Be Cleared are removed from the grid element. Dividing cells undergo cell division during which a new daughter cell is generated and enters the local population. When the grid element contains fewer than the carrying capacity of the grid element a new cell is cloned from the dividing cell. At this point the difference between Malignant and Normal cells is apparent in that Normal cells have Mutation and Invasion rates fixed at zero, whereas Malignant cells have non-zero values. In the case of Malignant cells, therefore, cloning can also incur a mutation in which one of the elements of the cell can change value, for example the Nutrient Target, a Gene Tolerance value or the cell Lifetime itself may undergo an increase or decrease. Note that the rate of mutation events is controlled by the Mutation Rate, which is itself mutable and can increase or decrease.

If the grid element is already at carrying capacity then the cell division process is more complex. In addition to undergoing a chance of mutation, Malignant cells may also undergo a migration event in which the cell moves into a randomly selected adjacent grid element. The rate of such migration events is controlled by the Invasion Rate, which, like the Mutation Rate, is mutable. Cells which are not selected for migration are added to the local population. To preserve the carrying capacity of the grid element, all cells are then ranked according to fitness and the least fit cells are removed. This ranked selection algorithm is not biased by cell type, and both Malignant and Normal cells are included in the process.

The fitness function, *F*, is designed to penalise cells which are poorly adapted to the *local* grid environment rather than being a global function across the entire population of cells. It is defined as:

(5) \begin{eqnarray*}F= \frac{1}{G} \sum _{g=1}^{G}{e}^{- \left( \left\vert {T}_{g}-{A}_{g} \right\vert /{T}_{g} \right) }\end{eqnarray*}

where *T* is the Gene Target and *A* is the Gene Factor value for each Gene in the Genome *G*. The fitness function yields a value in the range [0, 1], with a perfect fitness equal to 1, and is used as the ranking value when selecting the least fit cells for elimination. The rationale is that at any given point the cell that has Nutrient and Gene Factor demands which correspond most closely to the available levels in the local grid is the most well-adapted and hence the fittest. Note that this is evaluated at every clock tick, and therefore fitness changes over time as the local conditions change.

### Evolutionary strategies

The processing of Nutrient and Gene Factors is controlled by the treatment strategy object active during that clock tick. This software component, coded in Java, enables the NEATG system to model multiple evolutionary strategies, each of which can implement different algorithms in terms of controlling the rate of cellular attrition, ageing and division. Strategies are coded as Java components which extend an AbstractTreatmentStrategy class, which in turn implements an IStrategy Java interface. Dependency injection is used to load the required strategy, specified in a run-time configuration file, when it is required. Treatment strategies become active at specific time points, either by activation at a specified generation or at a specified level of tumour growth. Once triggered a treatment strategy can remain active until the final generation or for a specified number of generations. There is also a default ‘no treatment’ strategy during the iterations before and after the ‘active’ strategy is in operation.

## Results

### Homeostasis

Before exploring the results for different tumour growth scenarios it is important to validate the behaviour of the system during homeostatic and non-tumour scenarios. Cells in this scenario are supplied with optimal Nutrient and Gene Factor values, ensuring that they are unstressed and in ‘good health.’ In the absence of tumour cells we would expect that the system will display homeostatic behaviour characterised by regular cellular turn-over as cells age and die, and that cell population size will fluctuate but remain relatively constant.

To represent this scenario a series of experiments were run using a 25 × 25 grid. Empirical testing had shown that a grid of this size was sufficient to provide illustrative results in a wide range of scenarios, including tumour growth scenarios in addition to homeostasis and other non-tumour growth scenarios. The optimum cell population for each grid was set at 5, with a population of 10 cells as the maximum carrying capacity. The Nutrient Target used was 10, with a Nutrient Rate of 1. The Nutrient input to each grid element was also set at 10, ensuring that at optimum population level each cell would receive a Nutrient input of 10/5 = 2. A genome of three identical genes was used:

\begin{eqnarray*}G=\{(5.0,1.0),(5.0,1.0),(5.0,1.0)\}. \end{eqnarray*}

The Gene Factor supplied to each grid element was set at {25.0, 25.0, 25.0} , to ensure that each cell received the Target value of 5.0.

**Figure 3:**
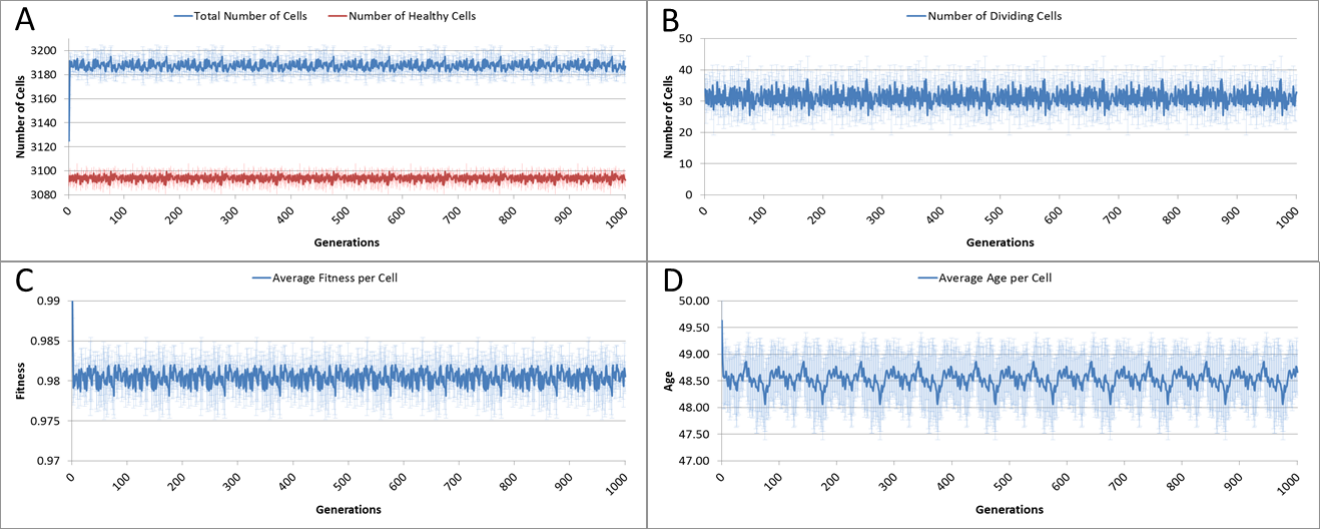
Cell change over time. (A) Total and healthy cell counts over time. (B) Number of dividing cells over time. (C) Average cell fitness over time. (D) Average cell age over time (all mean ± SD).

The system was run five times, with 1,000 iterations per run, and the results averaged for this analysis. Given our input parameters for a grid of 625 elements (25 × 25), and an optimum cell density of 5 cells per grid element, we would expect a total cell count of 3,125. However, not all of these cells will be healthy, some will be dividing or being cleared. Figure 3A shows the overall population of cells over time.

The number of dividing cells over time is shown in Fig. 3B. Note that the average over the 1,000 iterations is 31.25. This is as we would expect given that the Lifetime for the cells is 100, so that at any one time 1% of cells is dividing.

The average fitness, Fig. 3C, is high, fluctuating just below the maximum possible value of 1.0. And the average age, Fig. 3D, fluctuates just below a value of 50. These latter two figures display more clearly a pronounced periodicity which is also evident in the population density. This is due to the random distribution of ages in the initial cell population. In the absence of stress or environmental perturbation, the population of cells ages and divides in a uniform manner that preserves the distribution of ages from the initial population.

### Stress conditions

In the next experiments we assess the behaviour of NEATG when homeostasis is disturbed. In particular we are interested in the responses to changes in Nutrient and Gene Factors, as these both have an influence on cell ageing and survival. Again, this series of experiments does not include Malignant cells as we are primarily interested in exploring the behaviour of the system in non-tumour scenarios. For both of the following experiments, the same basic parameters as in the previous experiment are used.

**Figure 4:**
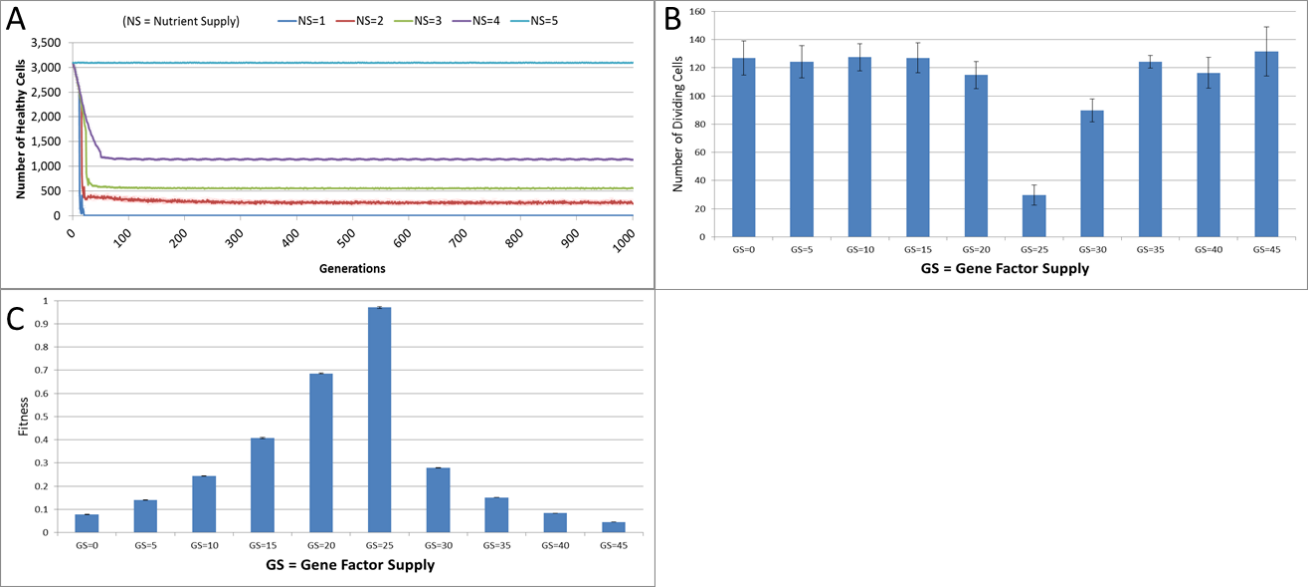
Changes during stress conditions. (A) Change in Healthy Cell count numbers in response to underfeeding. (B) Change in rate of cell division vs. Gene Factor Supply (at 1,000 generations). (C) Change in average fitness vs. Gene Factor Supply (at 1,000 generations) (all mean ± SD).

The first stress experiment varies the Nutrient input from 1 to 15, in integer steps. Given that the Nutrient Rate is set at a value of 1 and the optimum cell population is set to 5, we would expect that if the Nutrient Supply to each grid element falls below a value of 5 each cell in the grid would consume more nutrient than it receives as input and eventually deplete the value in its Nutrient Store (which was set to an initial value of 10). Figure 4A shows the number of healthy cells for different Nutrient Supply values. There is a decline in cell numbers over time for Nutrient Supply values below 5 but none for greater values (Fig. S1). Cell populations are therefore shown to be sensitive to the supply of Nutrient such that under-feeding can deplete numbers and in some cases ‘starvation’ reduces cell numbers to zero.

The supply of Gene Factors is the other external input to each grid element. These are analogous to generic growth and survival factors and are used to assess the health or otherwise of each cell in a grid element. In this experiment the same parameters are used as before, but the Gene Factor Supply is varied from {0.0, 0.0, 0.0} to {45.0, 45.0, 45.0}, in increments of 5.0.

There was little variation in cell counts in response to changes in Gene Factor Supply (Fig. S1); however, Gene Factors did have an influence on cell turnover, such that it was lowest for optimum values of Gene Factor Supply and increased by a factor of four as the deviations from the optimum values increased, as shown in Fig. 4B. The number of dividing cells at the optimal Gene Factor Supply value is around 1% of the total cell count, whereas for non-optimal Supply values there is an increased rate of cell division. This is as we would expect given that ‘unhealthy’ genes cause an increased rate of cell aging.

In addition to being a factor in the cellular aging process, the Genes are also used in calculations of cell fitness. Fitness is used in the rank selection process to identify the least fit cells when the population density in a grid element exceeds the maximum capacity. In this experiment no Malignant cells are present therefore the rank selection procedure is not active; however we can still assess the influence of the Gene Factor Supply on cell fitness, (which is defined in the range [0, 1]), as shown in Fig. 4C.

### Tumour growth—no treatment

Having established the behaviour of the system under homeostatic and non-tumour stress scenarios, we can now begin to introduce Malignant cells. Initially, we will explore the behaviour of NEATG in the absence of any treatment scenarios.

In this first series of experiments we will continue to use the same parameters as before, although the iteration period is increased to 2,000 to allow greater time for the evolution of appreciable number of tumour cells. Tumour growth is initiated by the insertion of a single Malignant cell into the grid element in the centre of our 25 × 25 grid. The only difference between this Malignant cell and the Normal cells is that the cell type is set to Malignant, and that it has a mutation rate of 5% and an invasion rate of 10%. Estimates of mutation rates in higher eukaryotes, including human cells, vary considerably. These initial values were derived from empirical testing of NEATG and were selected as they generated consistent tumour growth. In subsequent experiments, these values will be varied so that we can see how tumour growth patterns are affected. Drake et al. (1998) report a mutation rate per effective genome per replication of 0.004 in humans, which is an order of magnitude lower than the rate used here.

**Figure 5:**
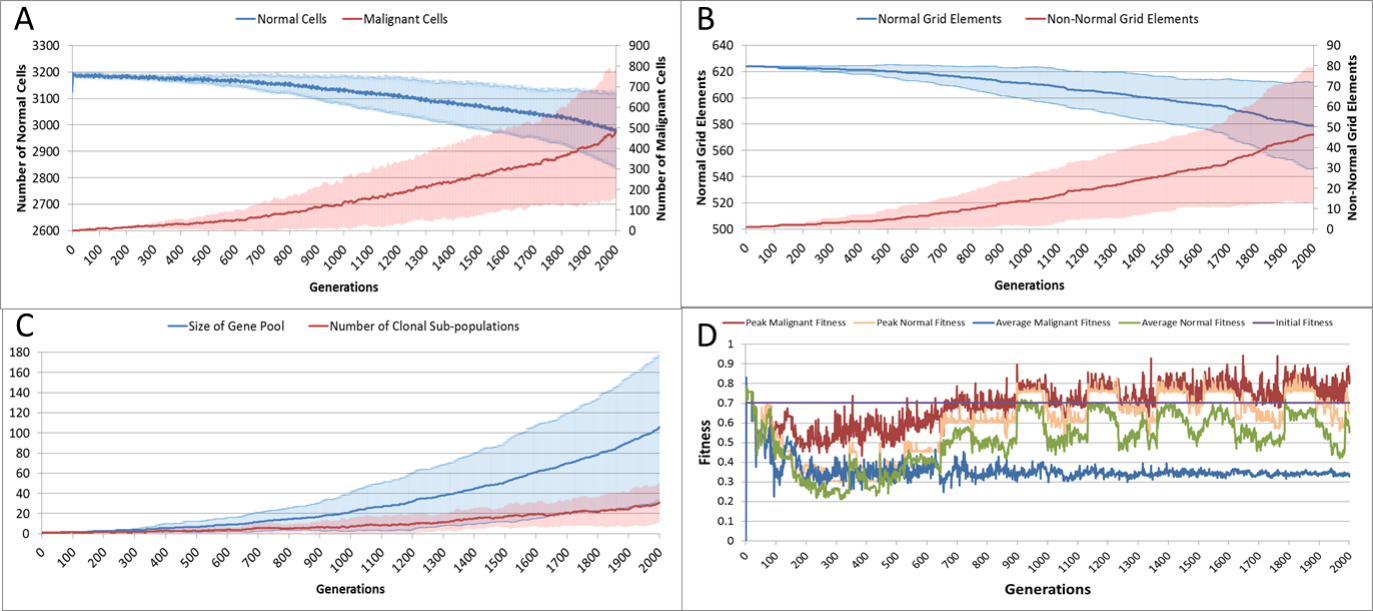
Tumour growth—no treatment. (A) Change in Normal and Malignant cell counts (Mean ± SD). (B) Change in Normal and Non-Normal Grid Element Counts (all mean ± SD). (C) Change in Gene Pool and Clonal Populations Over Time (all mean ± SD). (D) Change In Fitness Over Time (Mean).

With the introduction of Malignant cells we can view results both in terms of the changes in cell populations across the whole system and also in the evolution of the grid elements. The change in the global population counts cells is shown in Fig. 5A and grid elements in Fig. 5B.

Using Fig. 5A we can see that the doubling time for Malignant cells is approximately 500 generations. The doubling time for the NCI-60 human cancer cell lines range from 14.4 h (HCT-116 colon cancer) to 79.5 h (HOP-92 non-small cell lung cancer), with a median value of 33.25 h (National Cancer Institute, 2015). If we use this median value then each generation equates to 4 min, and the entire run of 2,000 generations is equivalent to around 5.5 days of *in vitro* growth.

Changes in grid elements and cell populations are not the only metrics of interest. Also of interest is the process of evolutionary change in the Malignant cell populations. In the initial population there is only a single genotype, but as shown in Fig. 5C the rate of change of the gene pool—the cumulative total of all clonal sub-populations which have been generated, including extinct populations in addition to existing ones—rises over time, increasing in line with the Malignant cell counts. Also shown in Fig. 5C is the rise in the number of clonal sub-populations, reflecting the growth of different active Malignant cell sub-populations.

The evolution of fitness is shown in Fig. 5D. The first Malignant cell has the same fitness as the Normal cells in the grid element into which it is inserted, however as the number of cells increases, the number of mutations rises, Malignant cells proliferate into neighbouring grid elements and competition for Nutrient and Gene Factors takes place. The noisy signals indicate a good deal of change and adaptation taking place over time. The initial high fitness value is degraded once the cell populations start to increase and competition takes place. It is also clear that the Normal cell population retains an average fitness that is higher than the average of the Malignant cell population. One plausible explanation is that many of the mutations are deleterious and do not lead to improved survival for those cells. However, if we look at the maximum values for the Malignant cells we can see that there are indeed some cells which do achieve a higher fitness than maximum of the Normal cells.

**Figure 6:**
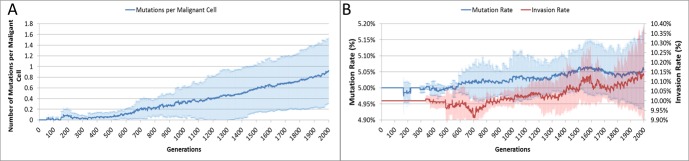
Mutation rates over time. (A) Mutations per Malignant cell over time. (B) Change in Mutation and Invasion Rates over time (all mean ± SD).

The average number of mutations per Malignant cell is shown in Fig. 6A. As can be seen for the first 100 generations or so there are no mutations, which accords with Fig. 5D.

The mutation rate and the invasion rate, which are both mutable characteristics show some change over time, as shown in Fig. 6B. While initially there is little change, indeed both rates dip below the starting values, both rates show an increasing trend over time.

**Figure 7:**
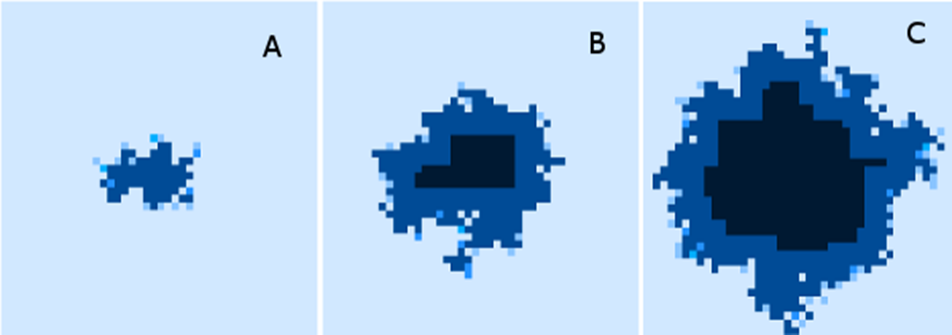
Spatial distribution of tumour growth. Evolving tumour mass at (A) 2,000 generations. (B) 4,000 generations. (C) 6,000 generations. Note that black areas are necrotic grid elements.

Finally, while we have explored the rates of change at the cellular and grid element levels, we have not explored the spatial distribution of the spread of Malignant cells. A representative example of the ‘no treatment’ scenario is shown in Fig. 7, an extended run of 6,000 generations and a grid size of 45 × 45 has been used to illustrate more fully the development of the tumour over time. Figure 7A shows ‘tendrils’ of cancer cells infiltrating into healthy tissue (light coloured background representing Normal cells) from the centre of the dark blue tumour mass, in Fig. 7B the tumour mass has expanded considerably and shows a black inner necrotic core and a perimeter of tumour cells with tendrils extending into the healthy cells. Finally Fig. 7C shows continued expansion, including an expanding area of necrosis. If allowed to continue expanding, the tumour eventually dominates the grid completely until further growth is impossible and the mass becomes mainly necrotic.

**Figure 8:**
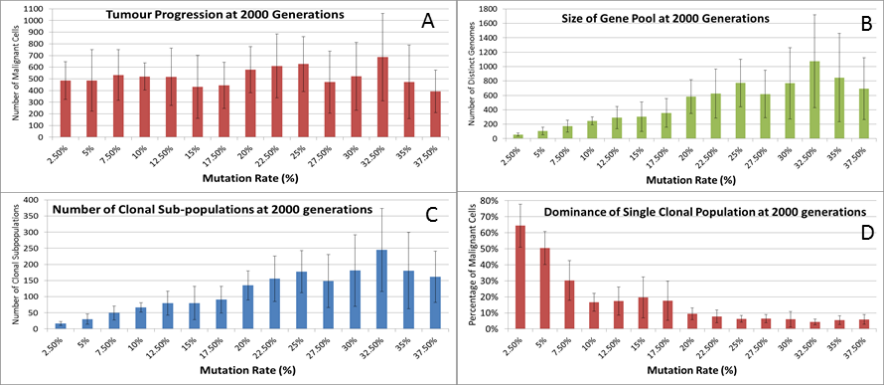
Mutation rates and clonal sub-populations. (A) Number of Malignant Cells. (B) Size of Gene Pool. (C) Number of Clonal Sub-populations. (D) Dominance of Single Clonal Population (all at 2,000 generations, mean ± SD).

We can vary the Mutation and Invasion rates to understand the impact they have on tumour growth. First we vary the Mutation Rate from 2.5% to 30% in increments of 2.5%, all other settings are as before. Note that while figures are shown for the final time point of 2,000 generations, these values are representative of the trends apparent at earlier time points. Whether we look at tumour progression in terms of grid elements or in terms of Malignant Cell counts, as in Fig. 8A, there is no direct relationship between mutation rate and tumour progression.

We would expect to see a correlation between the mutation rate and the size of the Gene Pool, Fig. 8B, though even here the relationship is not completely monotonous as a mutation rate of 32.5% generated a larger gene pool than a mutation rate of 37.5%. Similarly, if we look at the number of currently existing clonal sub-populations, Fig. 8C, there is a correlation with the mutation rate, but again this is not linear. Another interesting metric is the degree of dominance of the largest of the clonal sub-populations, Fig. 8D. This shows the percentage of the total number of Malignant cells which belong to the largest clonal sub-population and shows that a lower mutation rate yields a greater degree of dominance by a single clonal sub-population.

**Figure 9:**
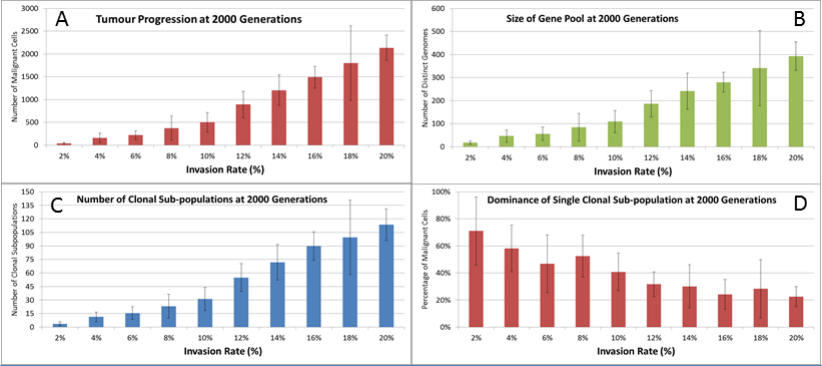
Invasion rates and clonal sub-populations. (A) Number of Malignant cells. (B) Size of Gene Pool. (C) Number of Clonal Sub-populations. (D) Dominance of Single Clonal Populations (all at 2,000 generations, mean ± SD).

We also vary the Invasion Rate to see what impact this has on the degree of tumour growth and the size of the gene pool. In this experiment the Invasion Rate is varied from 2% to 20% in 2% increments, the Mutation Rate of 5% is used; all other settings are as before. Clearly, as shown in Fig. 9A, there is a direct relationship between the Invasion Rate and the rate of tumour growth. More migration events correlate closely with increased tumour spread.

This increased rate of tumour growth also leads to an increase in the size of the Gene Pool and the number of clonal sub-populations, Figs. 9B and 9C. However, when compared to the scale of the increase of the Gene Pool with a rising Mutation Rate (Fig. 8B) it is clearly lower and indicates a less heterogeneous Malignant cell population. In terms of the dominance of a single clonal population, Fig. 9D, a lower Invasion rate is associated with an increased dominance by a single clonal sub-population, but even at a high Invasion Rate of 20% the degree of dominance is much higher than that associated with a high Mutation Rate (Fig. 8D).

### Tumour growth—with treatment

The previous experiments have shown that in the absence of any interventions the number of Malignant cells and Tumour grid elements increase over time. In the next series of experiments we investigate the impact on these growth patterns of a number of interventions using an active treatment strategy. This is loosely based on the example of high-dose cytotoxic chemotherapy. Just as with cytotoxic chemotherapy this is not a targeted therapy—it is applied to both Normal and Malignant cells. Where real chemotherapy causes apoptotic or necrotic cell death in rapidly dividing cells, the treatment strategy in this model flags cells above a specified age with the cell state of TO_BE_CLEARED. The arbitrary age cut-off is based on the value of a cell’s clock and this value is a configurable parameter. By adjusting the cut-off value we can approximately control the ‘toxicity’ of the treatment, the higher the cut-off value the more toxic the treatment as more cells will be flagged for disposal. The system also allows a degree of specificity in that we can make Malignant cells more susceptible to the treatment than Normal cells.

In this experiment, the same parameters will be used as in the No Treatment scenario. The treatment will commence at generation 1,500 (of 2,000), and will be applied for 25 generations. Three different toxicity values are assessed, with both Malignant and Normal having the same cut-off values. The values used are 0, 10 and 20, which means that any cell with a clock value ≤ the cut-off is ‘treated.’ Note that the zero cut-off value does not trigger cell division as in the default scenario, but triggers apoptosis and cell clearance. It represents the least toxic scenario and is therefore close to the ‘no treatment’ scenario.

**Figure 10:**
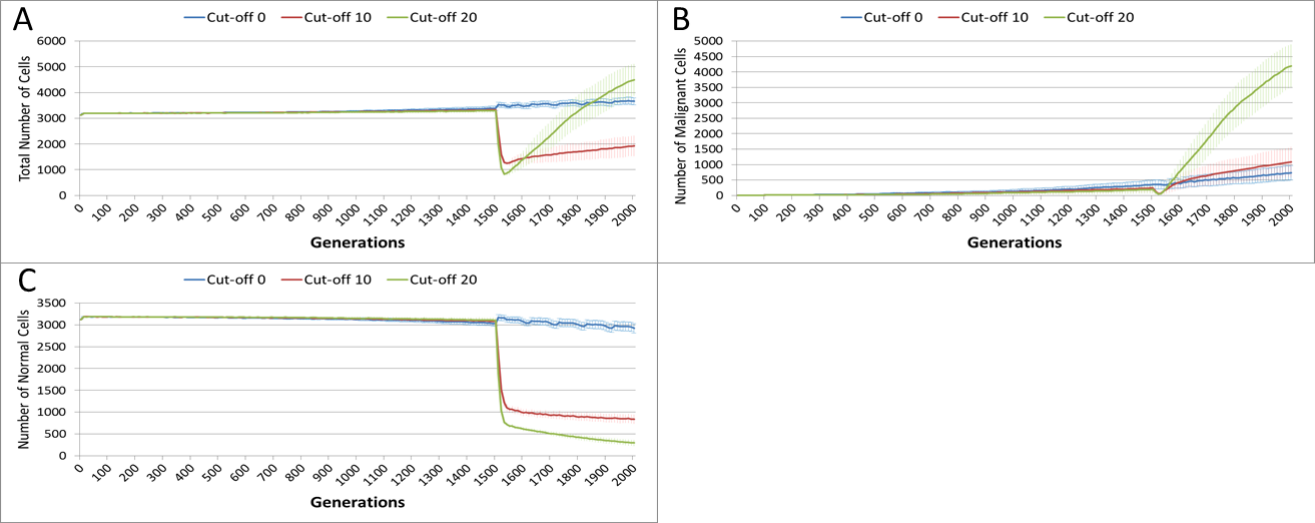
Tumour response to treatment toxicity. Treatment toxicity is altered by varying the cut-off of cell age below which cells are affected by cytotoxic treatment (e.g., Cut-off = 10 means all cells with a clock value ≤10 are flagged for removal). (A) Change in total cell numbers vs. toxicity. (B) Change in Malignant cell numbers vs. toxicity. (C) Change in Normal cell numbers vs. toxicity (Mean ± SD for all).

The effect of treatment on the total cell count, Fig. 10A, is dramatic. In the case of the more toxic treatments, there is a sharp decline in total cell numbers followed by a recovery, and in the case of the highest cut-off value of 20 cell growth accelerates above the pre-treatment trend.

**Figure 11:**
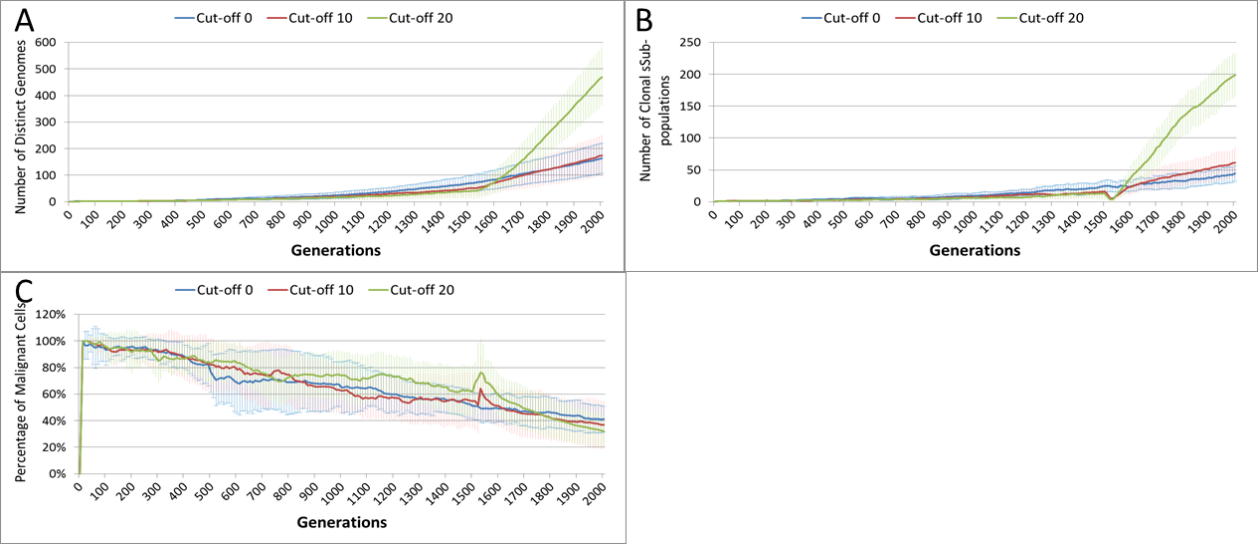
Treatment toxicity and clonal sub-populations. Treatment toxicity is altered by varying the cut-off of cell age below which cells are affected by cytotoxic treatment (e.g., Cut-off = 10 means all cells with a clock value ≤10 are flagged for removal). (A) Change in total cell numbers vs. toxicity. (B) Change in Malignant cell numbers vs. toxicity. (C) Change in Normal cell numbers vs. toxicity (Mean ± SD for all).

This growth trajectory is also reflected in the Malignant cell view of tumour growth, Fig. 10B. This shows that the slow rise in number is briefly interrupted when treatment begins but then accelerates sharply after the completion of treatment. Furthermore in both figures the more aggressive treatment is related to an increased tumour growth rate following the cessation of treatment. The change in the Normal Cell population is shown in Fig. 10C. The treatment induces a sharp reduction in cell numbers that continues even after the cessation of treatment, though not at the same rate. We can assume that the decline in Normal cell numbers has provided the conditions in which Malignant cells can expand rapidly in number. Supporting evidence is provided by the Gene Pool trends, shown in Fig. 11A. Here we can see that following treatment there is an increase in the size of the Gene Pool, indicating a post-treatment burst of clonal evolution.

The number of active clonal subpopulations, Fig. 11B, shows a similar trend—a slow increase until treatment commences at which point there is a dip in numbers followed by a post-treatment evolutionary explosion. Another view of this evolutionary burst, Fig. 11C, shows that the process of tumour growth leads to an increase in genetic heterogeneity, as measured by the decreasing proportion of the Malignant cell population belonging to the largest sub-population. The increasing heterogeneity is interrupted when treatment begins and there is a spike which shows that the largest sub-population increases as a proportion of the total, from which we can infer that a number of clonal sub-populations have been exterminated completely, in line with Fig. 11B.

In clinical practice maximum tolerated dose (MTD) chemotherapy does not cause equal levels of damage to all cell populations. Because it impacts rapidly proliferating cells the ‘collateral damage’ to non-tumour cells is restricted to certain populations of non-cancer cells in the immune system, gut and other tissues associated with the side effects of treatment (Chen et al., 2007). We can model this differential impact in the NEATG system by setting a lower cut-off value for Normal cells compared to Malignant cells, thus causing fewer Normal cells to be affected. In the following experiment the cut-off for the Normal cells is set to 10, and for the Malignant cells it is set to 15, 20 and 25 in three different scenarios. All other parameters are the same as in the previous experiment.

**Figure 12:**
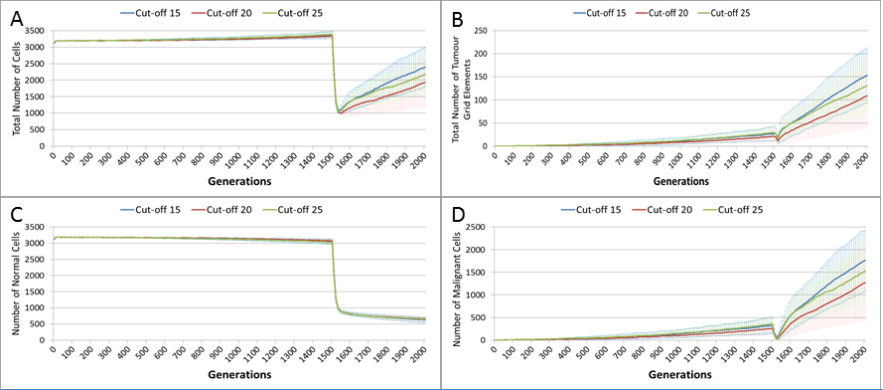
Tumour response to differential treatment toxicity. Differential treatment toxicity is modelled by varying the cell age below which cells are affected by cytotoxic treatment (e.g., Cut-off = 10 means all cells with a clock value ≤10 are flagged for removal) and applying different cut-off values for Normal and Malignant cells. Normal cell toxicity is fixed at cut-off = 10. (A) Change in total cell numbers vs. differential toxicity. (B) Change in Malignant cell numbers vs. differential toxicity. (C) Change in Normal cell numbers vs. differential toxicity (Mean ± SD for all).

In terms of the total cell counts, Fig. 12A, there is a similar pattern to the previous experiment, although the rate of recovery is much lower than in Fig. 10A. The lower sensitivity of the Normal cells means that even when the cut-off for the Malignant cells matches the previous values, the recovery of cell populations is lower. The pattern of increased tumour growth and evolutionary change following the cessation of treatment also occurs, Fig. 12B.

The lower sensitivity of the Normal cells does not mean that they are immune from effects of treatment. Figure 12C shows a marked decline when treatment commences, followed by a continued decline after treatment ends. Note there is no difference in the three scenarios shown, indicating that the Normal cells are not affected directly by the higher sensitivity of the Malignant cells. The values shown here are a close match to those shown for the Cut-off 10 scenario illustrated in Fig. 10C.

Two rather obvious questions arise from this data. The first is what happens if the period of treatment is extended? It is clear that for the duration of treatment the number of Malignant cells, tumour grid elements and clonal populations decrease. Is it possible to extend the treatment period so that the entire Malignant cell population is destroyed? Secondly, it is clear that the treatment damages Normal cells and that this coincides with increased cancer growth following the cessation of treatment. Therefore we can ask what happens in the case when the differential toxicity is such that there is *no* damage to the Normal cells—in other words what would happen in the case of a ‘magic bullet’ which has toxic effects only on Malignant cells? These questions are addressed in turn in the next two of experiments.

In the following experiment the treatment duration which was varied from 15–60 generations, in increments of 5. A differential toxicity was used, with a Malignant cut-off value of 20 and a Normal value of 10, all other settings are unchanged.

**Figure 13:**
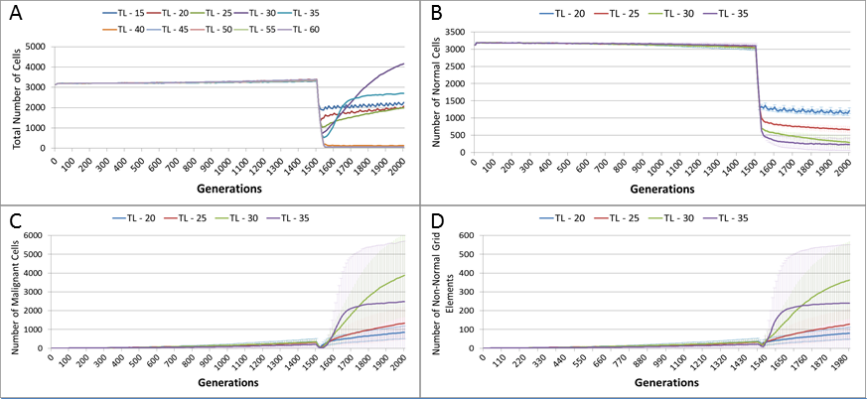
Tumour response to treatment length. TL, Treatment Length (number of generations in which treatment is active). (A) Total cell count vs. treatment length (mean only). (B) Normal cell count vs. treatment length (mean ± SD). (C) Malignant cell count vs. treatment length (mean ± SD).

Figure 13A, shows a relationship between the treatment length and the size of the total cell population. The relationship is complex and non-linear, but it is apparent that treatment duration longer than 40 generations causes significant reductions in the total population. This result was robust to repeated runs of the system and there was essentially no difference between results for any treatment length above this level. Furthermore, this upper cut-off figure for treatment length was related to the length of the cell Lifetime (which is 100 in these experiments). In order to simplify the exposition, the rest of the results in this experiment will focus on treatment lengths of 20–35.

The effect of treatment length on the Normal and Malignant cell populations is shown in Figs. 13B and 13C respectively. In the case of the Normal cell populations increasing treatment length is strongly associated with the scale of the decline in cell numbers. However, in the case of the Malignant cells, the treatment length is also associated with the rate of recovery. Figure 13C shows that longer treatment length can sometimes lead to an accelerated increase in Malignant cell numbers, though for treatment lengths beyond 40 (data not shown), there is no recovery in cell numbers, (as should be clear from the collapse in total cell counts in Fig. 13A). The somewhat surprising result is that in some cases a more aggressive treatment (longer treatment period) can lead to an unexpected acceleration in tumour growth.

**Figure 14:**
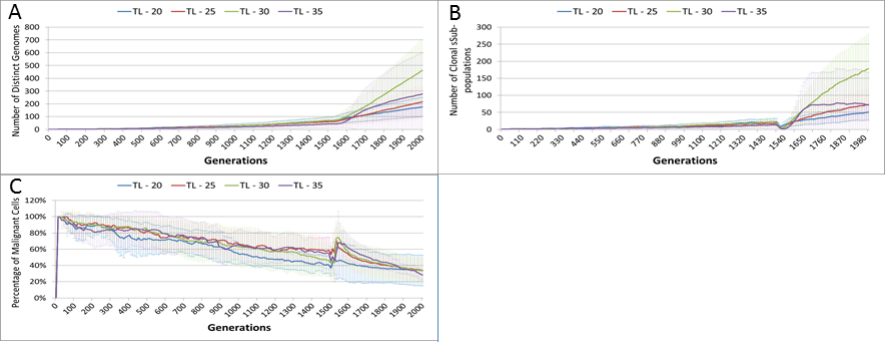
Treatment length and clonal sub-populations. TL, Treatment Length (number of generations in which treatment is active). (A) Size of Gene Pool vs. Treatment Length. (B). Number of clonal sub-populations vs. Treatment Length. (C) Sub-clonal Population Dominance vs. Treatment Length (Mean ± SD for all).

Length of treatment is also associated with an increase in the size of the Gene Pool, Fig. 14A, and acts as a spur to clonal evolution, as shown in Fig. 14B.

A further indication of the effect of treatment length on clonal evolution is shown in Fig. 14C, which charts the percentage of the total Malignant population in the most populous clonal sub-population. It is clear that longer treatment increases dominance as cells from less popular genotypes are removed, whereas for the short treatment of 20 generations there is no such spike in dominance.

**Figure 15:**
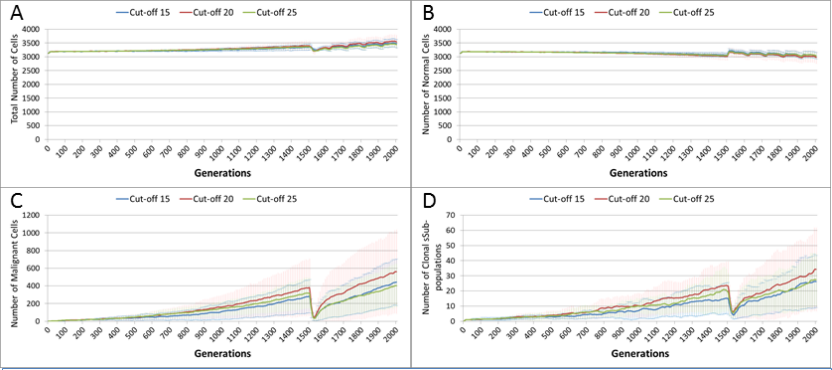
Tumour response to ‘magic bullet’. Treatment toxicity is altered by varying the cut-off of cell age below which cells are affected by cytotoxic treatment (e.g., Cut-off = 10 means all cells with a clock value ≤10 are flagged for removal). Here we model ‘no collateral damage’ and toxicity only applies to Malignant cells. (A) Total cell count vs. no collateral damage. (B) Normal cell count vs. no collateral damage. (C) Malignant cell count vs. no collateral damage. (D) Clonal sub-populations vs. no collateral damage (Mean ± SD for all).

In the final experiment in this section we investigate a ‘magic bullet’ scenario where treatment is applied only to Malignant cells. In this experiment three different toxicity levels are applied to the Malignant cells, representing cut-off values of 15, 20 and 25. In stark contrast to Figs. 10A and 12A, treatment does not lead to a sharp decline in total cell numbers, as shown in Fig. 15A. This is confirmed by the Normal cell numbers, Fig. 15B, where there is a slow decline prior to the commencement of treatment followed by a recovery in numbers and then a slow decline again.

The impact of treatment on Malignant cells, Fig. 15C, shows that the increase in cell numbers is reversed sharply by the treatment but is then followed by a recovery and a resumption of tumour growth. However, note that while the pattern is similar to previous experiments, the absolute number of Malignant cells is markedly lower than in Figs. 10B and 12B.

In terms of the impact on clonal evolution, Fig. 15D, while there is a pause during the treatment period, it continues at a similar rate to the pre-treatment trend afterwards. Again, while this pattern is familiar, the number of clonal sub-populations is lower than in previous experiments, as shown by Figs. 11B and 14B.

## Discussion

The results outlined above display a range of behaviours and phenomena which are indicative of real tumour growth. In the first instance the model is capable of reproducing homeostatic behaviour. In optimal conditions the model displays a steady turnover of cells, which age and divide in such a manner that the target cell population is preserved. However, under stress conditions, such as a restriction in the Nutrient supply or a reduction in Gene Factors, we see a change in behaviour. In the case of underfeeding or starvation we see that cell numbers are markedly reduced; however, over-feeding does not lead to an increase in cell populations.

For Gene Factors, we see that under or over-supply does not impact cell numbers to the same extent, though both scenarios lead to a small reduction in total cell numbers. The variations in Gene Factor supply do however impact on cell turnover, with an increase in rates of cell division in both under and over-supply situations. In this respect we may view the impact of deviations from the Gene Factor target values acting as mitogenic factors. There is also a marked impact on the calculation of cell fitness, with deviations from the optimal values fitness. We may conclude, therefore, that variations in the Gene Factor supply are deleterious to some extent, but do not cause the same level of cellular damage as restriction in the supply of Nutrient.

### Tumour growth

Once tumour growth is initiated the proliferation of cancer cells, (also reflected in the number of affected Grid Elements), increases in the absence of any counter-measures (i.e., left untreated). As each Grid Element can support a number of cells over and above the optimum level, this initial increase in numbers does not displace or replace non-cancer cells. However, once the carrying capacity of the Grid Element has been reached there is a competition between cells in which ultimately the Malignant cells out-compete the Normal cells. The influence of carrying capacity on Malignant cell growth is illustrated in Fig. 17B, which shows that changing the trigger point for competition by varying the optimum cell count has an impact on the rate of tumour growth. Over time the number of Malignant cells increases and the rate of invasion increases, while there is a corresponding decrease in Normal cell numbers. As with the homeostatic case, this behaviour is not pre-programmed but emerges from the interactions between the cells, between neighbouring Grid Elements and the operation of a few simple rules. Additionally, there is a consistent increase in the number of clonal sub-populations as growth continues—mirroring the genetic heterogeneity which is a hall-mark of real tumour growth (Sun & Yu, 2015). The system also shows that in the face of changing conditions there is an increase in the number of clonal sub-populations and a decrease in the dominance of the most populous sub-clone over time, again, reflecting real tumour genetic heterogeneity (Jamal-Hanjani et al., 2015).

We should note that in the first instance the seeded Malignant cell has the same genomic structure as the Normal cell population in these experiments; that is, the Malignant cell is not conferred any genetic advantage over the rest of the non-Malignant cell population. The single difference between the Malignant cell and the Normal cell is that the Malignant cell is flagged as such and that it has an ability to mutate and undergo repeated division. It may be assumed that the increasing success of the Malignant cells in outcompeting Normal cells may be due to an increasing evolutionary fitness that arises through a succession of mutational events occurring during cell division. However, the data does not support this assumption.

Evolutionary fitness is not defined in absolute or global terms in NEATG. Instead it is a local function that reflects cellular adaption to the changing conditions *in each Grid Element*. Thus it is clear from the data, as shown in Fig. 5D, that in general the fitness of many Malignant cells is lower than the initial fitness of the Normal cells, and that it often decreases as a result of intra-Grid Element competition between cells. Furthermore, many mutations are actually deleterious and do not confer evolutionary advantage over competing cells, Normal or Malignant. Some Malignant cells do experience mutations which provide an advantage, and these are the cells which manage to survive and expand in number. However, a cell with a positive advantage in one Grid Element may migrate to an adjacent Grid Element and find that it is less fit and therefore does not survive. This view of evolutionary fitness as locally responsive to the environment and therefore having an impact on the success, or otherwise, of genetic mutations is in line with more recent theoretical models of evolutionary processes in cancer (Rozhok & DeGregori, 2015).

The rate of evolutionary change is initially set by the Mutation Rate, which is heritable and mutable. It may be thought that the Mutation Rate would be an important driver in the rate of cancer growth; however our data show that in this model it has a weak influence on the rate of growth of cancer. It does however directly influence the size of the Gene Pool and the number of clonal sub-populations. More influential in terms of driving growth is the Invasion Rate, which represents the probability that a dividing Malignant cell in an overcrowded Grid Element can migrate to a neighbouring Grid Element. The data show that this is a very strong driver of growth rates, but it does not lead to the same increase in the size of the Gene Pool or the number of clonal sub-populations. These observations are in line with results reported by Enderling, Hlatky & Hahnfeldt (2009) also using an agent-based model of tumour growth.

In terms of modelling interventions against tumour growth, we have explored the use of a treatment option that loosely mimics maximum tolerated dose chemotherapy in two key respects. Firstly, the treatment is not genetically targeted—it applies to both Normal and Malignant cells, though we can confer an increased sensitivity to Malignant cells if required. Secondly, the treatment induces cell death in affected cells, analogous to the apoptotic or necrotic cell death induced by chemotherapy. Finally, cells are affected depending on where they are in the cell cycle—which is modelled in this instance by the reading of the cell clock.

### Tumour regrowth

One of the most interesting emergent behaviours exhibited by the NEATG system is the response of the modelled tumour mass to a treatment that mimics aspects of chemotherapy treatment.

**Figure 16:**
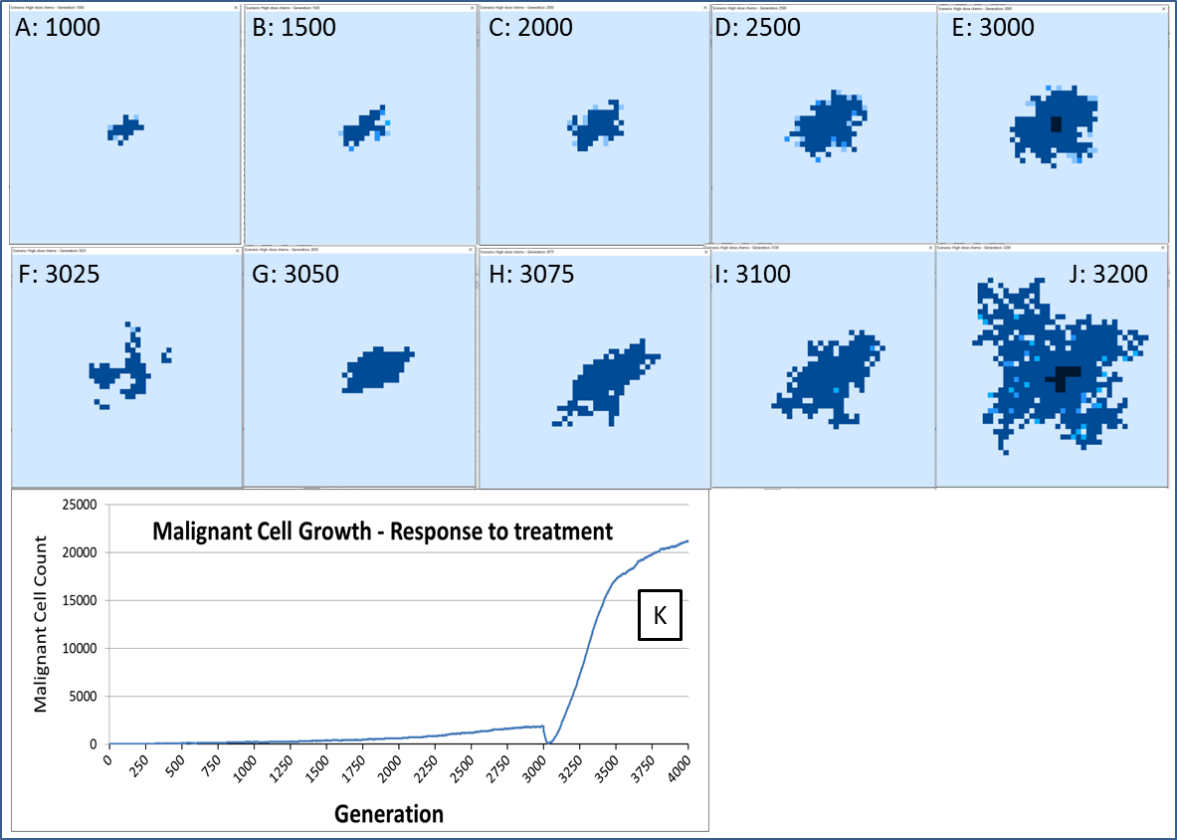
Growth of tumour mass over time. Treatment is initiated at 3,000 generations (E). (F) and (G) show tumour mass shrinkage. (H–J) show the accelerated growth following treatment. (K) shows the corresponding graph of malignant cell counts over time.

The response to this treatment, which we have varied in intensity and duration, is consistent in our experiments. There is an initial response marked by massive tumour kill followed by a resumption of tumour growth, which is often characterised by an accelerated and aggressive tumour expansion, as shown in Fig. 16.

**Figure 17:**
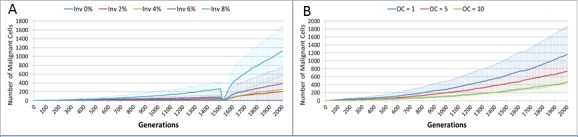
Invasion rate and optimal cell count. (A) Tumour growth for different levels of Invasiveness. (B) OC, Optimal Cell Count—cell competition is initiated when the number of cells in a grid element reaches the OC level (Mean ± SD for all).

This response to treatment bears some resemblance to real cancer treatment, where an initial reduction in tumour growth, characterised as complete or partial remission, is followed by renewed tumour growth or the appearance of metastatic disease. Clinically this phenomenon is sometimes termed accelerated repopulation (Davis & Tannock, 2000; Kurtova et al., 2015; Yom, 2015). While the mechanisms of treatment resistance in real tumours are complex and multifactorial, it is assumed that tumour heterogeneity is an important factor; a tumour may harbour clonal subpopulations which are resistant to treatment and which therefore benefit from reduced competition after chemo-sensitive populations have been destroyed by treatment (Von Manstein et al., 2013; Gottesman et al., 2016).

In the NEATG model, treatment resistance is not related to drug efflux or other mechanisms of acquired resistance. Instead the phenomenon is associated with a pool of cells which survive due to their age (i.e., they are above the treatment cut-off age) and which are therefore faced with a decreased level of competition for resources and a lower population density of cells in each Grid Element. This is a finding in line with the Norton-Simon hypothesis in which a proportion of cancer cells in tumour are resistant not due to biochemical factors but due to the growth kinetics of the tumour (Norton & Simon, 1977).

Increasing the intensity or duration of treatment as a strategy to improve response is shown to be problematic in that it can cause reductions in Normal cell numbers which do not recover and therefore this strategy is assumed to be deleterious. Again, there is a clear parallel to clinical experience in which increased toxicity causes excess morbidity without necessarily leading to improved outcomes.

### The role of mutations

The rule of genetic mutation is a central concern in oncology, both in terms of fundamental theories and increasingly at a clinical level in terms of targeted treatments. At a simplistic level the SMT places the delinquent cell at the centre of cancer development, whereas the TOFT places the poor neighbourhood central to the story (Baker, 2014; Sonnenschein et al., 2014). A key difference between these competing theories is the role of cellular proliferation. The SMT suggests that in the non-transformed state cells are non-proliferative by default. Mutations in genes associated with cell cycle control mean cells become proliferative and malignant. In contrast the TOFT posits that cells are proliferative by default and that this proliferative ability is kept in check at the tissue level. A disordered tissue results in the removal of the proliferative blocks and the cell can multiply without control.

In our model, both cell and tissue (Grid Element) level structures are featured. The process of cancer initiation consists of seeding a transformed cell into a grid element and letting it proliferate. The model does not have anything to say about how the initial cell is transformed, it is taken as a given. The initial cell has the same parameters as the untransformed cells; the only difference is that proliferative blocks have been removed. The transformed cell, and its progeny, is able to accumulate mutations during cell division and replication. Some of these mutations will be deleterious and some will be advantageous, we would expect therefore that the average fitness of the Malignant population will increase and that these advantageous mutations will drive further evolutionary change—particularly mutations that increase the Invasion rate. However, this does not appear to occur. Indeed, a surprising result is that neither the Mutation Rate nor the Invasion Rate, which are both heritable and mutable, appears to undergo significant increase during the process of tumour growth. In fact, as shown in Fig. 6B, both show marginal rates of change, and can rise and fall rather than rising monotonically and driving malignant growth. While some mutations may provide evolutionary advantage, it is clear that the majority of mutations are passenger mutations rather than driver mutations. This is another instance where the NEATG model parallels biological systems, as it has become increasingly clear that the majority of somatic mutations in human tumours are also passenger mutations, many of which are actively deleterious to the cancer cell (Greenman et al., 2007; McFarland et al., 2013; McFarland, Mirny & Korolev, 2014).

The question arises then as to whether mutational change is a necessary precondition for cancer growth in this model. To investigate this question an additional series of experiments was performed in which the Mutation Rate was set at zero, and the Invasion Rate varied from zero to 8% in increments of 2%, with all other settings as in the previous set of experiments. The results show that Malignant cell growth can occur even with a zero Mutation rate, which was verified by confirming that the Gene Pool retained a constant value of 1 (Fig. S2). This may be viewed as analogous to tissue hyperplasia where non-transformed cells proliferate at an increased rate. The rate of growth in this model, as shown in Fig. 17A, depends on the Invasion Rate, as one would expect, but even at the lowest non-zero rate tumour growth occurs, and furthermore the growth rate accelerates after treatment.

What is more, the data shows that with a zero rate of Invasion and Mutation there is growth in Malignant cell numbers to the maximum possible in the Grid Element where seeding occurred, but that without an Invasion Rate there is no possibility of a Malignant cell migrating to a neighbouring Grid Element. One implication of this result is that in the NEATG model cancer growth is not driven primarily by somatic mutation and is primarily dependent on proliferation and invasiveness.

**Figure 18:**
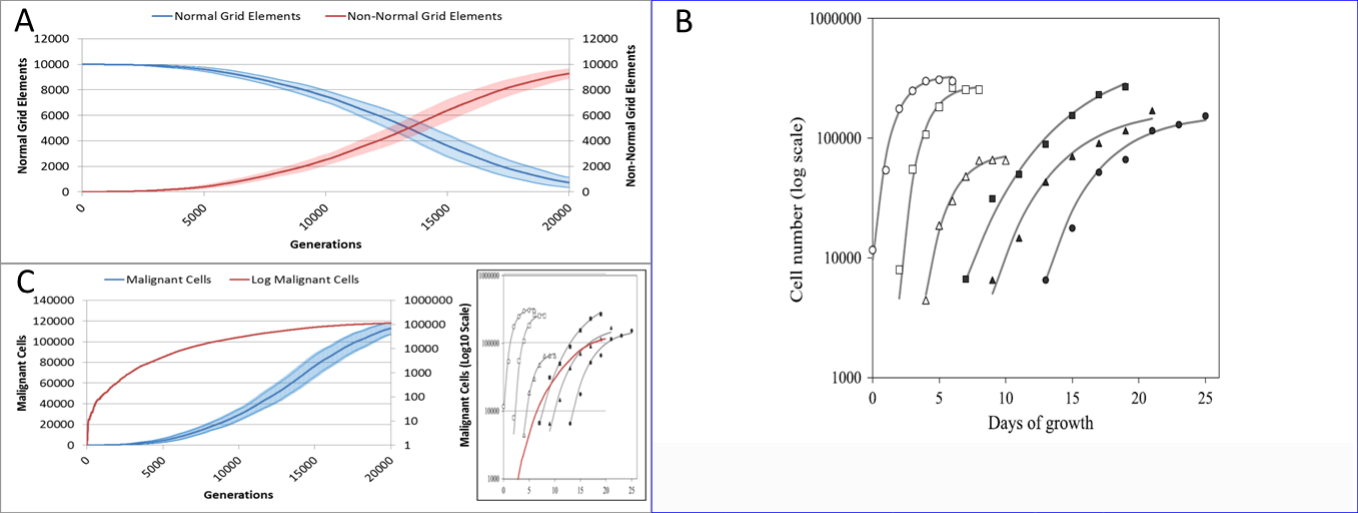
Comparison to real tumour growth. (A) Change in Normal and Non-Normal Grid Element Counts (mean ± SD). (B) Growth of monolayer cells from human cancer cell lines (reproduced from Castro et al., 2003). The solid lines are the best-fit Gompertzian curves and the points are experimental data of the cell panel: SW-620 (○), HT-29 (□), U-251 (△), NCI-H520 (●), NCI-H596 (■) and A-549 (▴). The cell-density scale is transformed logarithmically. (C) Change in Malignant cell numbers—absolute (mean ± SD) and log transformed (mean) Inset shows NEATG mean Malignant cell growth (red line) to scale compared to human cancer cell lines.

### Reflecting on real tumour growth

Clearly this is a very simple model that does not incorporate many biologically relevant oncogenic mechanisms—the model was deliberately designed to be as parsimonious as possible. Validation of the growth curves produced by this model in comparison to biologically relevant systems is not straightforward but we can make some preliminary assessments. As NEATG is a two-dimensional grid model the most appropriate place to look for validation is *in vitro* monolayer systems using a range of different cancer cell lines. To fully assess that this is the case with the NEATG model a series of additional experiments were performed in which the model grid was scaled up and the run-time, in terms of the number of generations, was significantly extended by a factor of 10. Running NEATG with a grid size of 100 × 100 and for 20,000 generations allowed for a more realistic generation of cell numbers and tumour growth, (albeit at the cost of increased computation time).

The first thing to note is that the growth of the tumour area more clearly follows a sigmoidal curve, as shown by looking at the growth curves in Fig. 18A. These results can be compared to the log growth curves in cell numbers for a range of human colon carcinoma, malignant glioma and non-small cell lung carcinoma cell lines reported by Castro et al. (2003), and reproduced in Fig. 18B. We can compare these results to the log transformed growth curve of Malignant cells, Fig. 18C, and verify that there is a similar pattern of growth, and that the asymptotic cell numbers are of roughly the same order of magnitude. Furthermore we can compare the *x*-axes and make an informal estimate that in these experiments 1,000 generations in NEATG roughly corresponds to one day of growth in the monolayer systems used by Castro et al. in their experiments. In doing so, we can scale the NEATG Malignant cell growth line appropriately and compare to the human cancer cell lines growth, as shown in the inset in Fig. 18C.

While this falls short of fully validating the growth patterns produced by NEATG, they do indicate that the growth rates that emerge from model share some quantitative and qualitative features of the growth of human cancer cell lines *in vitro*.

Yet, given the limited physiology modelled by the system it has reproduced a series of emergent phenomena which are analogous to biologically relevant phenomena—tumour growth, intra-tumour genetic heterogeneity, response to virtual cytotoxic intervention and accelerated repopulation.

By definition this is an evolutionary model, ‘descent with modification’ is a given, but as we have seen it is also possible to run the model with a zero mutation rate and still generate a growing population of Malignant cells. One notes that although they harbour no mutations and may be considered Normal cells with a hyperplastic phenotype, there are also rare instances of cancers in which no genetic mutations or epigenetic drivers are present (Versteeg, 2014). We have also defined Malignant cells as those with the ability to mutate and to move into neighbouring Grid Elements. How these abilities arise is not a question we are investigating in the model. What then are the key drivers of tumour growth and accelerated repopulation? The detailed analysis of the behaviours outlined in the Results suggests that there are two key drivers:

∙ Cell competition
∙ Cell death.

Competition occurs in the NEATG model within each Grid Element when the population density reaches a set level (the optimum cell count). As can be seen in Fig. 17B, when competition begins earlier (when the optimum cell count is 1), the rate of tumour growth is much higher. As one would expect, competition also spurs growth of the gene pool (Fig. S3). Competition for resources leads to cell death when the number of cells exceeds the carrying capacity of the Grid Element. A ranked selection algorithm means that the least fit (within that Grid Element) cells are removed. Importantly, this competitive process takes place entirely within a Grid Element and is a process that involves both cell-to-cell (cell-autonomous) and tissue-level (non-cell-autonomous, defined by the optimum cell count for the Grid Element) factors.

Cell death arises both from the competition between cells within each Grid Element and also exogenously via ‘treatment’—in this work loosely modelled on maximum tolerated dose chemotherapy. It is clear from the data that increasing the rate of cell death, both in Normal and Malignant cells, leads to accelerated repopulation and more aggressive tumour growth.

So, while both cell competition and cell death are both integral components of the model, the *impact* that these have is not pre-programmed. These are unexpected key drivers of the emergent behaviours that the model displays and the major findings of this work.

Many of the *core* findings from molecular biology are *not* included in this model. For example the NEATG model does not explicitly make use of the cancer stem cell hypothesis. Cancer stem cells (CSC), also known as tumour-initiating or cancer-initiating cells, are functionally characterised as a small fraction of tumour cell populations with the ability to self-renew, differentiate into multiple cell types and to generate new tumours when transplanted (Reya et al., 2001; Jordan, Guzman & Noble, 2006; Bozorgi, Khazaei & Khazaei, 2015). Crucially, CSC are assumed to generate the non-CSC cells which make up the major population of malignant cells in a tumour. In addition to being characterised by a range of cell markers (CD44+, CD133+, ALDH1 etc.), CSC are theorised to be relatively chemo- and radio-resistant and a key factor in resistance to treatment (Yang & Rycaj, 2015).

However, the CSC hypothesis is increasingly being challenged as evidence emerges that rather than being a distinct cell population there is a set of properties which together define ‘stemness’ (Lewis, 2008; Antoniou et al., 2013; Wang et al., 2015). In particular, the claim that tumour growth is mainly attributable to the rapid proliferation of CSC populations rather than the non-CSC fraction is open to some dispute (Adams & Strasser, 2008; Hegde et al., 2012). Additionally there is evidence that cancer cells display a significant degree of plasticity such that ‘stemness’ traits can be acquired by non-CSC cells (Chaffer et al., 2011; Cabrera, Hollingsworth & Hurt, 2015). Indeed, some recent work suggests that non-CSC cells acquire stem-like properties in response to therapeutic challenge with chemotherapy (Hu et al., 2012; Martins-Neves et al., 2016).

NEATG, therefore, does not explicitly model CSC and non-CSC populations but makes the simplifying assumption that all Malignant cells are proliferative. The key point is that the existence of CSC, whether as a separate population of cells or a collection of cellular traits, is immaterial to the operation of the model and the ability to reproduce tumour cell growth. At this level of abstraction, the behaviour of the model would be the same regardless of the underlying complexities of the CSC hypothesis.

Similarly, the model does not include oncogenes, specific molecular pathways, a realistic cell cycle, a vascular or lymphatic system, immune responses, different cell types, tumour stroma and many more biologically important aspects of real disease. However, the model does propose that cell competition and cell death have an important, and perhaps underestimated, role in patterns of tumour growth and response to treatment. Given that induction of cell death, particularly via the apoptotic pathway, is central to the most common forms of cancer treatment this would be of some clinical significance if confirmed in the laboratory. Based on these results, it is therefore hypothesised that cell competition and cell death are major drivers of tumour growth.

There are some indications that these two aspects of cancer biology are of biological significance.

A number of investigators have looked at the question of the role of cell competition in cancer, for example Baker & Li (2008), and Moreno (2008), both referring to results from research in *Drosophila melanogaster*which outlined the process whereby cells of differing genotype *within* a given compartment engage in competition such that *locally* less fit cells undergo apoptosis and are replaced with *locally* fitter cells. Very recent work by Suijkerbuijk et al., (2016) has described the process whereby cell competition between APC-/- intestinal adenoma cells and normal host cells in *Drosophila melanogaster* leads to cell death in normal cells, host tissue attrition and the invasion of more rapidly proliferating adenoma cells. Eichenlaub and colleagues have also investigated cell competition in the same animal model (Eichenlaub, Cohen & Herranz, 2016). They report that EGFR over-expression in wing imaginal disc cells leads to benign tissue hyperplasia and subsequent epithelial tumour formation.

We should note that there are different cell types, molecular drivers and pathways active in the latter two studies, yet both groups report that blocking the apoptotic process blocks tumour development. This prompts the conclusion that targeting cell competition itself may be a valid strategy in cancer therapy (Gil & Rodriguez, 2016).

While cell competition may be a necessary pre-condition of cancer development, it is not sufficient, and our model clearly indicates that cell death is also required. This poses the question as to the role of cell death, particularly apoptosis, in tumour growth. One of the hallmarks of cancer is defined as ‘resistance to apoptosis’ (Hanahan & Weinberg, 2011), yet it is known that tumours show a high rate of apoptosis, and at least in some cancer types high apoptosis rates are a negative prognostic factor (Nishimura et al., 1999). A number of recent studies have outlined the much more complex relationship between cancer and apoptosis than has been assumed in the past (Gregory & Pound, 2011; Wang et al., 2013; Labi & Erlacher, 2015; Lauber & Herrmann, 2015; Ford et al., 2015). While these studies outline numerous mechanistic explanations as to why increased apoptosis may lead to increased tumour growth, it is clear that there are underlying phenomena which may have important clinical implications in terms of treatment strategies.

One rather obvious conclusion is that rather than aiming at maximum tumour kill using traditional cytotoxic chemotherapy perhaps, other treatment strategies which produce lower levels of cancer cell death may be more beneficial. For example, using metronomic chemotherapy, in which chemotherapeutic drugs are administered at non-cytotoxic doses and with no treatment breaks is one such strategy (Scharovsky, Mainetti & Rozados, 2009; Kareva, Waxman & Klement, 2014; André, Carré & Pasquier, 2014). Another example is the concept of ‘adaptive therapy,’ in which chemotherapy is used to maintain a population of tumour cells rather than aiming to maximise tumour kill (Gatenby et al., 2009; Enriquez-Navas et al., 2016).

While it is clear that the NEATG system does not provide us with mechanistic explanations for the pro-tumour growth effects of cell competition and apoptosis, it does direct our attention to these areas of current, active but not yet mainstream research. Having directed our attention to the role of cell competition and cell death, there is ample scope for continuing to use the model to explore the processes at work and, perhaps, to suggest relevant laboratory experiments in light of further model results.

## Conclusion

Having identified cell competition and apoptosis as key concerns, we may look to incorporate additional aspects of this in more detail. For example, the onset of cell competition is triggered when the optimum cell count is reached. In part this is a function of the carrying capacity of the Grid Element—when this level is exceeded, Malignant cells are able to migrate to a randomly selected neighbouring Grid Element (a stochastic process depending on the Invasion Rate). In some respects this is analogous to tissue stiffness or rigidity in that Grid Elements can be made more or less ‘stiff’ by increasing or decreasing the carrying capacity. Tissue stiffness is also a current concern in oncology (Wei & Yang, 2016) and may be amenable to additional investigation by extending this model.

NEATG has been designed as a platform for investigating different interventions and how they impact the growth of Malignant cells and tumour Grid Elements. In the experiments described in this paper only one strategy, loosely based on maximum tolerated dose chemotherapy, has been explored. Clearly there is scope for additional interventions to be modelled; for example, combinations of Nutrient restriction and chemotherapy, a treatment strategy of some clinical interest (Raffaghello et al., 2008; Safdie et al., 2009; Lee et al., 2012), may be modelled in NEATG. Similarly, the use of metronomic chemotherapy, chemo-switch strategies, targeted therapies and the use of different treatment schedules are also amenable to modelling using the NEATG system.

The value of agent-based evolutionary models is that they can generate biologically relevant behaviour through algorithmic means, which may in turn shed new light on the underlying biological systems. Obviously increasing the complexity of the model so that additional features are included may be of some value. Here we have generated hypotheses as to the role that cell competition and cell death have in cancer, suggesting that these relatively under-researched processes may have much greater important than has hitherto been accepted. At the same time the model has not required the implementation of cancer stem cell populations, specific oncogenic pathways and has shown a limited role for genetic mutation—all of which are currently predominant concerns in much cancer research.

##  Supplemental Information

10.7717/peerj.2176/supp-1 Figure S1 Healthy cell counts did not show significant variation for different levels of Gene Factor

10.7717/peerj.2176/supp-2 Figure S2 The size of the Gene Pool is constant when the Mutation Rate is 0%. Variations in Invasion Rate do not impact the Gene Pool even during tumour growth

10.7717/peerj.2176/supp-3 Figure S3 Increased competition, with a lower value of OC (Optimum Cell Count), is associated with an increased size of Gene Pool

## Additional Information and Declarations

### Data Availability

The following information was supplied regarding data availability: Figshare: http://dx.doi.org/10.6084/m9.figshare.1614842;
http://figshare.com/articles/NEATG_Data_and_Code/1614842.

## References

[ref-1] Adams JM, Strasser A (2008). Is tumor growth sustained by rare cancer stem cells or dominant clones?. Cancer Research.

[ref-2] Allen M, Louise Jones J (2011). Jekyll and Hyde: the role of the microenvironment on the progression of cancer. The Journal of Pathology.

[ref-3] André N, Carré M, Pasquier E (2014). Metronomics: towards personalized chemotherapy?. Nature Reviews. Clinical Oncology.

[ref-4] Antoniou A, Hébrant A, Dom G, Dumont JE, Maenhaut C (2013). Cancer stem cells, a fuzzy evolving concept: a cell population or a cell property?. Cell Cycle.

[ref-5] Baker SG (2014). A cancer theory kerfuffle can lead to new lines of research. Journal of the National Cancer Institute.

[ref-6] Baker NE, Li W (2008). Cell competition and its possible relation to cancer. Cancer Research.

[ref-7] Barcellos-Hoff MH, Lyden D, Wang TC (2013). The evolution of the cancer niche during multistage carcinogenesis. Nature Reviews. Cancer.

[ref-8] Basanta D, Simon M, Hatzikirou H, Deutsch A (2008). Evolutionary game theory elucidates the role of glycolysis in glioma progression and invasion. Cell Proliferation.

[ref-9] Bizzarri M, Cucina A (2014). Tumor and the microenvironment: a chance to reframe the paradigm of carcinogenesis?. BioMed Research International.

[ref-10] Bozorgi A, Khazaei M, Khazaei MR (2015). New findings on breast cancer stem cells: a review. Journal of Breast Cancer.

[ref-11] Cabrera MC, Hollingsworth RE, Hurt EM (2015). Cancer stem cell plasticity and tumor hierarchy. World Journal of Stem Cells.

[ref-12] Castro MAA, Klamt F, Grieneisen VA, Grivicich I, Moreira JCF (2003). Gompertzian growth pattern correlated with phenotypic organization of colon carcinoma, malignant glioma and non-small cell lung carcinoma cell lines. Cell Proliferation.

[ref-13] Chaffer CL, Brueckmann I, Scheel C, Kaestli AJ, Wiggins PA, Rodrigues LO, Brooks M, Reinhardt F, Su Y, Polyak K, Arendt LM, Kuperwasser C, Bierie B, Weinberg RA (2011). Normal and neoplastic nonstem cells can spontaneously convert to a stem-like state. Proceedings of the National Academy of Sciences of the United States of America.

[ref-14] Chen Y, Jungsuwadee P, Vore M, Butterfield DA, St Clair DK (2007). Collateral damage in cancer chemotherapy: oxidative stress in nontargeted tissues. Molecular Interventions.

[ref-15] Davis AJ, Tannock JF (2000). Repopulation of tumour cells between cycles of chemotherapy: a neglected factor. The Lancet. Oncology.

[ref-16] De Sousa E Melo F, Vermeulen L, Fessler E, Medema JP (2013). Cancer heterogeneity— a multifaceted view. EMBO Reports.

[ref-17] Drake JW, Charlesworth B, Charlesworth D, Crow JF (1998). Rates of spontaneous mutation. Genetics.

[ref-18] Eichenlaub T, Cohen SM, Herranz H (2016). Cell competition drives the formation of metastatic tumors in a drosophila model of epithelial tumor formation. Current Biology.

[ref-19] Enderling H, Hahnfeldt P (2011). Cancer stem cells in solid tumors: is “evading apoptosis” a hallmark of cancer?. Progress in Biophysics and Molecular Biology.

[ref-20] Enderling H, Hlatky L, Hahnfeldt P (2009). Migration rules: tumours are conglomerates of self-metastases. British Journal of Cancer.

[ref-21] Enriquez-Navas PM, Kam Y, Das T, Hassan S, Silva A, Foroutan P, Ruiz E, Martinez G, Minton S, Gillies RJ, Gatenby RA (2016). Exploiting evolutionary principles to prolong tumor control in preclinical models of breast cancer. Science Translational Medicine.

[ref-22] Fisher R, Pusztai L, Swanton C (2013). Cancer heterogeneity: implications for targeted therapeutics. British Journal of Cancer.

[ref-23] Ford CA, Petrova S, Pound JD, Voss JJLP, Melville L, Paterson M, Farnworth SL, Gallimore AM, Cuff S, Wheadon H, Dobbin E, Ogden CA, Dumitriu IE, Dunbar DR, Murray PG, Ruckerl D, Allen JE, Hume DA, Van Rooijen N, Goodlad JR, Freeman TC, Gregory CD (2015). Oncogenic properties of apoptotic tumor cells in aggressive B cell lymphoma. Current Biology.

[ref-24] Gatenby RA, Gillies RJ, Brown JS (2011). Of cancer and cave fish. Nature Reviews. Cancer.

[ref-25] Gatenby RA, Silva AS, Gillies RJ, Frieden BR (2009). Adaptive therapy. Cancer Research.

[ref-26] Gerlee P, Anderson ARA (2007). An evolutionary hybrid cellular automaton model of solid tumour growth. Journal of Theoretical Biology.

[ref-27] Gerlee P, Basanta D, Anderson ARA (2011). Evolving homeostatic tissue using genetic algorithms. Progress in Biophysics and Molecular Biology.

[ref-28] Gil J, Rodriguez T (2016). Cancer: the transforming power of cell competition. Current Biology.

[ref-29] Gillies RJ, Verduzco D, Gatenby RA (2012). Evolutionary dynamics of carcinogenesis and why targeted therapy does not work. Nature Reviews. Cancer.

[ref-30] Gottesman MM, Lavi O, Hall MD, Gillet J-P (2016). Toward a better understanding of the complexity of cancer drug resistance. Annual Review of Pharmacology and Toxicology.

[ref-31] Greenman C, Stephens P, Smith R, Dalgliesh GL, Hunter C, Bignell G, Davies H, Teague J, Butler A, Stevens C, Edkins S, O’Meara S, Vastrik I, Schmidt EE, Avis T, Barthorpe S, Bhamra G, Buck G, Choudhury B, Clements J, Cole J, Dicks E, Forbes S, Gray K, Halliday K, Harrison R, Hills K, Hinton J, Jenkinson A, Jones D, Menzies A, Mironenko T, Perry J, Raine K, Richardson D, Shepherd R, Small A, Tofts C, Varian J, Webb T, West S, Widaa S, Yates A, Cahill DP, Louis DN, Goldstraw P, Nicholson AG, Brasseur F, Looijenga L, Weber BL, Chiew Y-E, DeFazio A, Greaves MF, Green AR, Campbell P, Birney E, Easton DF, Chenevix-Trench G, Tan M-H, Khoo SK, Teh BT, Yuen ST, Leung SY, Wooster R, Futreal PA, Stratton MR (2007). Patterns of somatic mutation in human cancer genomes. Nature.

[ref-32] Gregory CD, Pound JD (2011). Cell death in the neighbourhood: direct microenvironmental effects of apoptosis in normal and neoplastic tissues. The Journal of Pathology.

[ref-33] Hanahan D, Coussens LM (2012). Accessories to the crime: functions of cells recruited to the tumor microenvironment. Cancer Cell.

[ref-34] Hanahan D, Weinberg RA (2011). Hallmarks of cancer: the next generation. Cell.

[ref-35] Hegde GV, De la Cruz C, Eastham-Anderson J, Zheng Y, Sweet-Cordero EA, Jackson EL (2012). Residual tumor cells that drive disease relapse after chemotherapy do not have enhanced tumor initiating capacity. PLoS ONE.

[ref-36] Hu X, Ghisolfi L, Keates AC, Zhang J, Xiang S, Lee D, Li CJ (2012). Induction of cancer cell stemness by chemotherapy. Cell Cycle.

[ref-37] Jalali R, Mittra I, Badwe R (2016). Cancer research: in need of introspection. The Lancet. Oncology.

[ref-38] Jamal-Hanjani M, Quezada SA, Larkin J, Swanton C (2015). Translational implications of tumor heterogeneity. Clinical Cancer Research.

[ref-39] Janes KA, Lauffenburger DA (2013). Models of signalling networks—what cell biologists can gain from them and give to them. Journal of Cell Science.

[ref-40] Jordan CT, Guzman ML, Noble M (2006). Cancer stem cells. The New England Journal of Medicine.

[ref-41] Kareva I (2011). What can ecology teach us about cancer?. Translational Oncology.

[ref-42] Kareva I, Waxman DJ, Klement GL (2014). Metronomic chemotherapy: an attractive alternative to maximum tolerated dose therapy that can activate anti-tumor immunity and minimize therapeutic resistance. Cancer Letters.

[ref-43] Krzeslak M, Swierniak A (2014). Four phenotype model of interaction between tumour cells. IFAC Proceedings.

[ref-44] Kurtova AV, Xiao J, Mo Q, Pazhanisamy S, Krasnow R, Lerner SP, Chen F, Roh TT, Lay E, Ho PL, Chan KS (2015). Blocking PGE2-induced tumour repopulation abrogates bladder cancer chemoresistance. Nature.

[ref-45] Labi V, Erlacher M (2015). How cell death shapes cancer. Cell Death & Disease.

[ref-46] Lauber K, Herrmann M (2015). Tumor biology: with a little help from my dying friends. Current Biology.

[ref-47] Lee C, Raffaghello L, Brandhorst S, Safdie FM, Bianchi G, Martin-Montalvo A, Pistoia V, Wei M, Hwang S, Merlino A, Emionite L, De Cabo R, Longo VD (2012). Fasting cycles retard growth of tumors and sensitize a range of cancer cell types to chemotherapy. Science Translational Medicine.

[ref-48] Lewis MT (2008). Faith, heresy and the cancer stem cell hypothesis. Future Oncology.

[ref-49] Martins-Neves SR, Paiva-Oliveira DI, Wijers-Koster PM, Abrunhosa AJ, Fontes-Ribeiro C, Bovée JVMG, Cleton-Jansen A-M, Gomes CMF (2016). Chemotherapy induces stemness in osteosarcoma cells through activation of Wnt/*β*-catenin signaling. Cancer Letters.

[ref-50] McFarland CD, Korolev KS, Kryukov GV, Sunyaev SR, Mirny LA (2013). Impact of deleterious passenger mutations on cancer progression. Proceedings of the National Academy of Sciences of the United States of America.

[ref-51] McFarland CD, Mirny LA, Korolev KS (2014). Tug-of-war between driver and passenger mutations in cancer and other adaptive processes. Proceedings of the National Academy of Sciences of United States of America.

[ref-52] Moreno E (2008). Is cell competition relevant to cancer?. Nature Reviews. Cancer.

[ref-53] National Cancer Institute (2015). Developmental theraputics program—cell lines in the in vitro screen. https://dtp.cancer.gov/discovery_development/nci-60/cell_list.htm.

[ref-54] Nishimura R, Nagao K, Miyayama H, Matsuda M, Baba K, Matsuoka Y, Yamashita H, Fukuda M, Higuchi A (1999). Apoptosis in breast cancer and its relationship to clinicopathological characteristics and prognosis. Journal of Surgical Oncology.

[ref-55] Norton L, Simon R (1977). Tumor size, sensitivity to therapy, and design of treatment schedules. Cancer Treatment Reports.

[ref-56] Pantziarka P (2015). Primed for cancer: Li Fraumeni Syndrome and the pre-cancerous niche. Ecancermedicalscience.

[ref-57] Poleszczuk J, Enderling H (2016). Cancer stem cell plasticity as tumor growth promoter and catalyst of population collapse. Stem Cells International.

[ref-58] Psaila B, Kaplan RN, Port ER, Lyden D (2007). Priming the “soil” for breast cancer metastasis: the pre-metastatic niche. Breast Disease.

[ref-59] Quail DF, Joyce JA (2013). Microenvironmental regulation of tumor progression and metastasis. Nature Medicine.

[ref-60] Raffaghello L, Lee C, Safdie FM, Wei M, Madia F, Bianchi G, Longo VD (2008). Starvation-dependent differential stress resistance protects normal but not cancer cells against high-dose chemotherapy. Proceedings of the National Academy of Sciences of the United States of America.

[ref-61] Reya T, Morrison SJ, Clarke MF, Weissman IL (2001). Stem cells, cancer, and cancer stem cells. Nature.

[ref-62] Ribba B, Alarcon T, Marron K, Maini PK, Agur Z (2004). *Cellular Automata*.

[ref-63] Rozhok AI, DeGregori J (2015). Toward an evolutionary model of cancer: considering the mechanisms that govern the fate of somatic mutations. Proceedings of the National Academy of Sciences of the United States of America.

[ref-64] Saetzler K, Sonnenschein C, Soto AM (2011). Systems biology beyond networks: generating order from disorder through self-organization. Seminars in Cancer Biology.

[ref-65] Safdie FM, Dorff T, Quinn D, Fontana L, Wei M, Lee C, Cohen P, Longo VD (2009). Fasting and cancer treatment in humans: a case series report. Aging.

[ref-66] Scharovsky OG, Mainetti LE, Rozados VR (2009). Metronomic chemotherapy: changing the paradigm that more is better. Current Oncology.

[ref-67] Silva AS, Gatenby RA (2010). A theoretical quantitative model for evolution of cancer chemotherapy resistance. Biology Direct.

[ref-68] Sonnenschein C, Soto AM, Rangarajan A, Kulkarni P (2014). Competing views on cancer. Journal of Biosciences.

[ref-69] Suijkerbuijk SJE, Kolahgar G, Kucinski I, Piddini E (2016). Cell competition drives the growth of intestinal adenomas in drosophila. Current Biology.

[ref-70] Sun X, Yu Q (2015). Intra-tumor heterogeneity of cancer cells and its implications for cancer treatment. Acta Pharmacologica Sinica.

[ref-71] Tian T, Olson S, Whitacre JM, Harding A (2011). The origins of cancer robustness and evolvability. Integrative Biology.

[ref-72] Versteeg R (2014). Cancer: tumours outside the mutation box. Nature.

[ref-73] Von Manstein V, Yang CM, Richter D, Delis N, Vafaizadeh V, Groner B (2013). Resistance of cancer cells to targeted therapies through the activation of compensating signaling loops. Current Signal Transduction Therapy.

[ref-74] Wang R-A, Li Q-L, Li Z-S, Zheng P-J, Zhang H-Z, Huang X-F, Chi S-M, Yang A-G, Cui R (2013). Apoptosis drives cancer cells proliferate and metastasize. Journal of Cellular and Molecular Medicine.

[ref-75] Wang T, Shigdar S, Gantier MP, Hou Y, Wang L, Li Y, Al Shamaileh H, Yin W, Zhou S, Zhao X, Duan W (2015). Cancer stem cell targeted therapy: progress amid controversies. Oncotarget.

[ref-76] Wei SC, Yang J (2016). Forcing through tumor metastasis: the interplay between tissue rigidity and epithelial-mesenchymal transition. Trends in Cell Biology.

[ref-77] Weinberg RA (2014). Coming full circle—from endless complexity to simplicity and back again. Cell.

[ref-78] Yang T, Rycaj K (2015). Targeted therapy against cancer stem cells. Oncology Letters.

[ref-79] Yom SS (2015). Accelerated repopulation as a cause of radiation treatment failure in non-small cell lung cancer: review of current data and future clinical strategies. Seminars in Radiation Oncology.

