# Supplementary figures and images for: Emergent properties of a computational model of tumour growth
Source: PeerJ. 2016 Jun 29;4:e2176. doi: 10.7717/peerj.2176 (PMC4933089; doi:10.7717/peerj.2176)

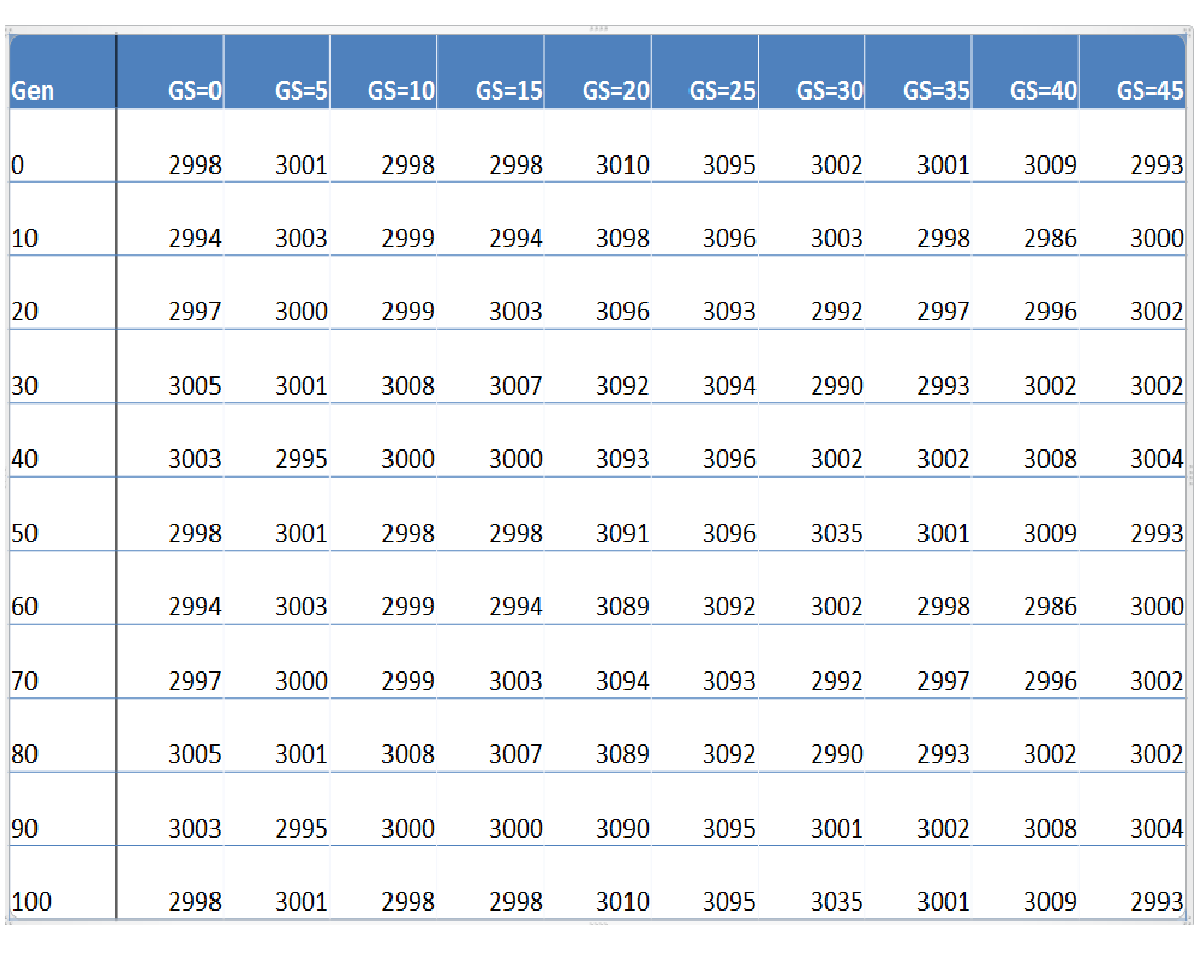

Supplement: Figure S1 [file peerj-04-2176-s001.png]

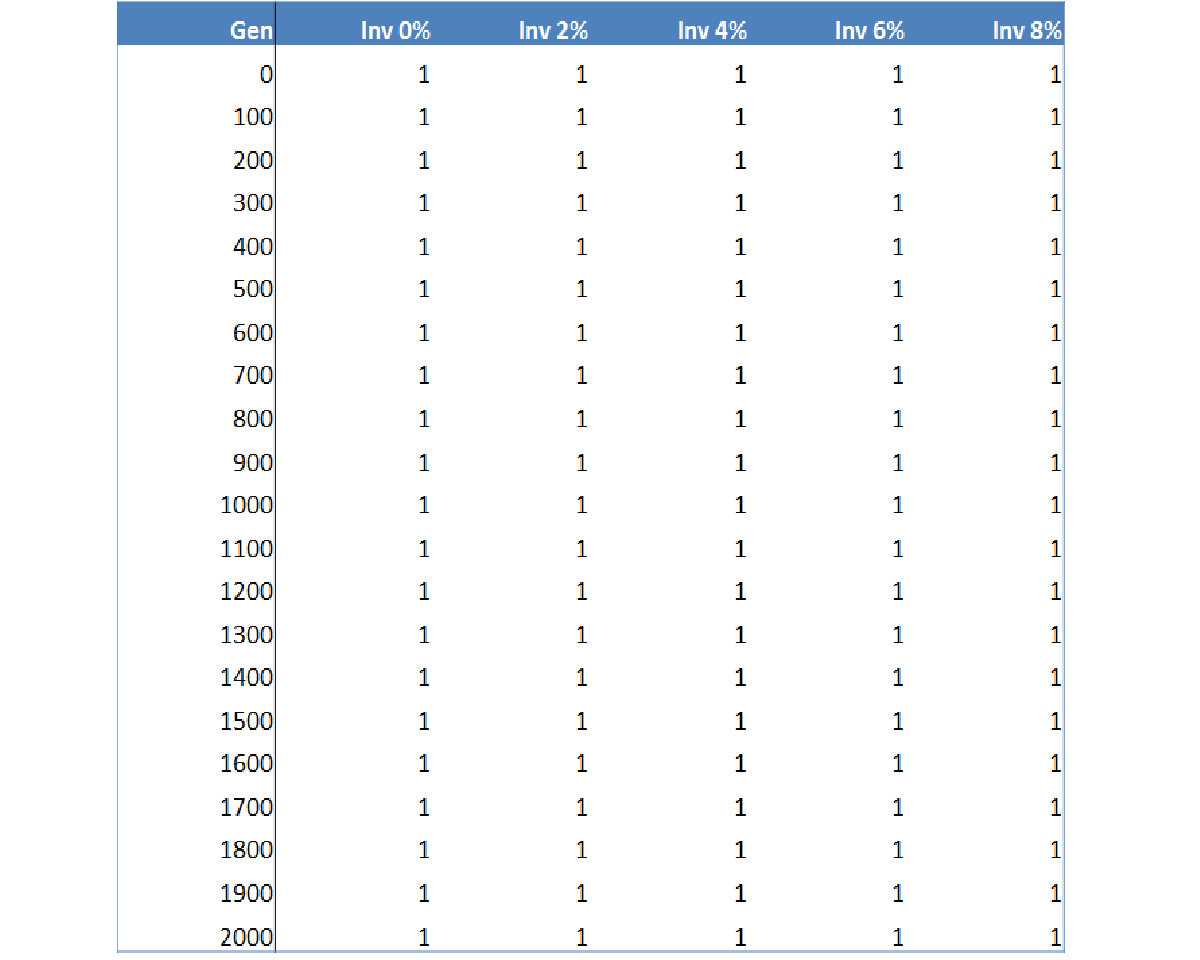

Supplement: Figure S2 [file peerj-04-2176-s002.png]

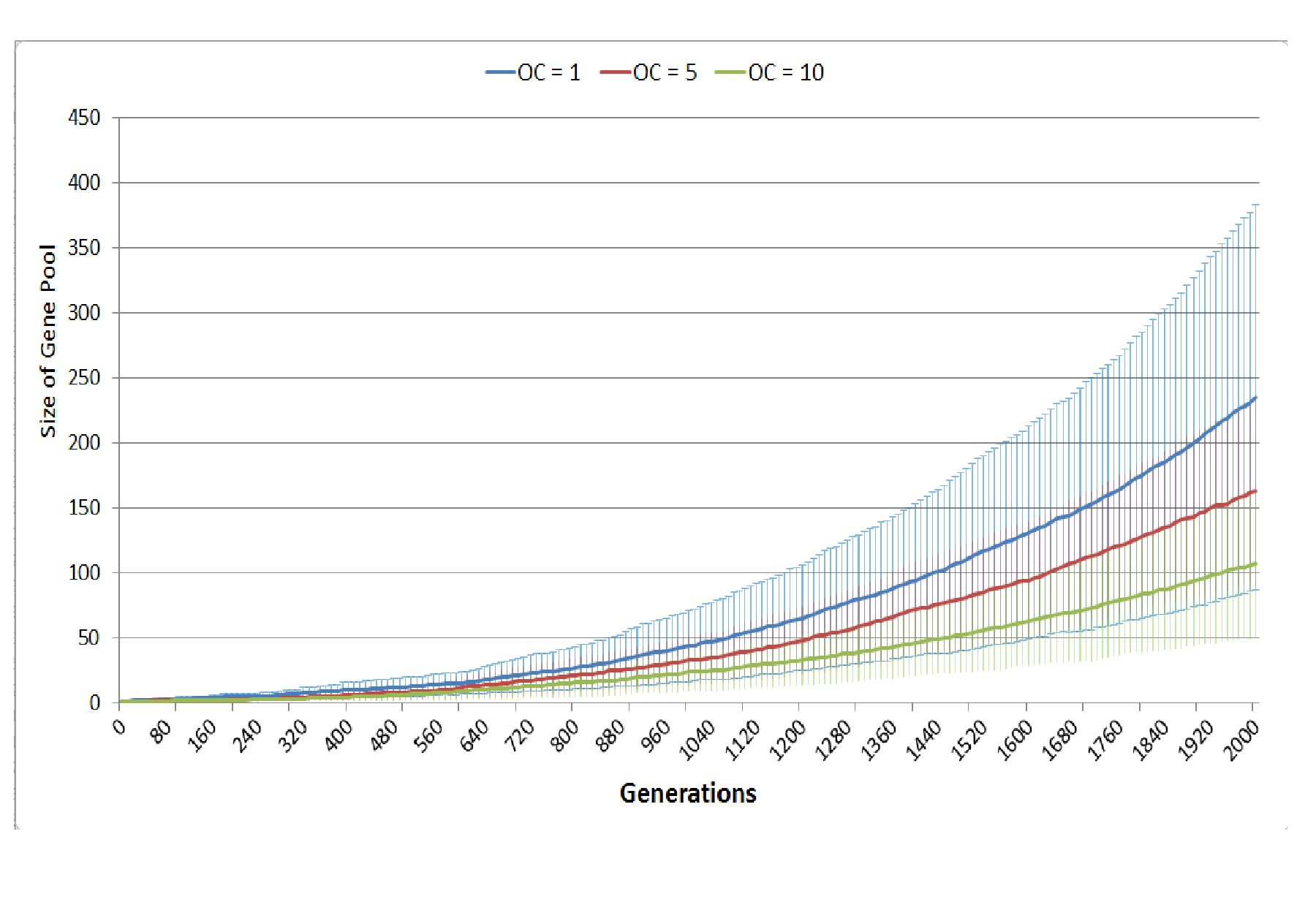

Supplement: Figure S3 [file peerj-04-2176-s003.png]
